# Effect of UK Quality and Outcomes Framework pay-for-performance programme on quality of primary care: systematic review with quantitative synthesis

**DOI:** 10.1136/bmj-2024-083424

**Published:** 2025-06-25

**Authors:** Leonard Ho, Stewart W Mercer, David Henderson, Eddie Donaghy, Bruce Guthrie

**Affiliations:** 1 NIHR Health Determinants Research Collaboration Aberdeen, Aberdeen, UK; 2 Advanced Care Research Centre, Usher Institute, University of Edinburgh, Edinburgh, UK; 3 Centre for Population Health Sciences, Usher Institute, University of Edinburgh, Edinburgh, UK

## Abstract

**Objective:**

To systematically review the one and three year impact on quality of care of the introduction and withdrawal of financial incentives in the UK Quality and Outcomes Framework pay-for-performance programme.

**Design:**

Systematic review with quantitative synthesis.

**Data sources:**

MEDLINE, Embase, CINAHL, PsycINFO, and Scopus databases were searched from 1 January 2004 to 3 September 2024.

**Study selection:**

Eligible studies used repeated cross sectional or cohort designs with consistent measurement before and after incentive introduction or withdrawal. Studies with a minimum of three time points before and after intervention were included in quantitative synthesis.

**Data extraction:**

Analysis used both reported impact if available and de novo interrupted time series analysis of extracted raw data if not reported by the original study.

**Data synthesis:**

Meta-analysis was not appropriate; findings were quantitatively synthesised by reporting medians and interquartile ranges of changes in quality or reported narratively. Risk of bias was assessed using the Mixed Methods Assessment Tool.

**Results:**

30 studies were included, with 11 providing data for quantitative synthesis. Across all indicators, evidence was found of improvement in recorded quality at one year after incentive introduction (83 indicators; median change compared with that predicted by pre-incentivisation trends 6.1 (interquartile range (IQR) 1.9 to 14.6) percentage points) but less consistently at three years (72 indicators; median change 0.7 (−2.1 to 8.9) percentage points). Impact was higher for process indicators with lower performance in the year before incentivisation. Incentive withdrawal was associated with reduction in recorded quality compared with predicted at both one year (31 indicators; median change −10.7 (IQR −17.9 to −3.8) percentage points) and three years (31 indicators; median change −12.8 (−21.0 to −4.4) percentage points). The largest changes with both incentive introduction and withdrawal were for complex process indicators such as diabetes foot screening, with smaller changes in simple processes such as blood pressure measurement, intermediate outcomes, and treatment indicators. For all types of indicators, the reduction in quality following incentive withdrawal generally matched or exceeded the gains observed after incentive introduction (for example, for 14 indicators with data for both, median change at three years for incentive introduction was a 1.4 (IQR −0.9 to 4.6) percentage point increase versus a 3.9 (IQR 2.2 to 11.6) decrease for incentive withdrawal).

**Conclusion:**

Quality and Outcomes Framework incentives consistently improved quality of care at one year beyond that predicted by pre-incentivisation trends, but by three years the impact was inconsistent and not clearly better than trend. Gains from incentivisation seemed to reverse after incentive withdrawal.

**Study registration:**

Prospero CRD42023467627.

## Introduction

“Pay for performance” is commonly deployed in healthcare internationally as a tool to improve quality of care. Although physicians and providers clearly respond to both explicit and implicit financial incentives, the effectiveness of pay for performance in improving quality remains uncertain.^1^ ^2^ ^3^ Pay for performance often seems to improve the recording of care processes, but evidence that introducing incentives improves intermediate outcomes (such as whether blood pressure is controlled) is inconsistent, with some studies showing benefit and others no benefit. Many studies also examine only short term (for example, one year) impact meaning that longer term effects are often uncertain.^1^ ^2^ Quality of care measured using quality indicators often worsens when incentives are withdrawn.^4^ ^5^ ^6^ For both introduction and withdrawal of incentives, distinguishing whether observed changes are due to better recording of care already being given or to changes in the care actually delivered is not straightforward. Variation in evidence of impact at least partly reflects heterogeneity in the design of evaluations, with weaker designs typically finding larger benefits of incentive introduction because they do not properly account for pre-incentivisation trends.^2^ ^7^

The UK Quality and Outcomes Framework (QOF) introduced across the National Health Service (NHS) in April 2004 remains one of the largest ever primary care pay-for-performance programmes in the world (supplementary box A). In its original form, it included more than 150 indicators and accounted for 20-25% of the income of UK primary care practices (which are typically small, physician owned organisations that contract to provide publicly funded NHS care). Over time, several indicators were retired and new indicators introduced, and the proportion of income tied to the QOF reduced. A large number of indicators were removed at the end of March 2014, and at the end of March 2016 NHS Scotland removed all QOF incentivisation. The QOF therefore provides a system-wide natural experiment to examine the impact of introducing and withdrawing financial incentives. The aim of this study was to systematically review studies of the impact of UK QOF incentives on quality of primary care, including quantitative synthesis of the one and three year impact of both incentive introduction and incentive withdrawal.

## Methods

### Study overview

We registered the protocol in PROSPERO (CRD42023467627) and report the review on the basis of the PRISMA (Preferred Reporting Items for Systematic Reviews and Meta-Analyses) guidelines.^8^ This paper reports findings for the first two registered review questions. (1) What was the impact on measured quality when incentives were introduced? (2) What was the impact on measured quality when incentives were withdrawn? For comparison we also report data for changes in indicators that were not incentivised, before and after introduction of the QOF in April 2004.

### Eligibility criteria

Studies on introduction and withdrawal of incentives were eligible if they used repeated cross sectional or cohort designs with consistent measurement of quality of care in defined UK populations before and after incentive introduction or withdrawal. We had two requirements for inclusion in the primary quantitative synthesis. Firstly, the measures examined had to be either the same as or very closely related to the incentivised indicator (for example, the QOF incentivised care for “diabetes” without specifying type, but we included data for the same quality indicators examined in people with type 1 diabetes) or directly related to it (for example, the QOF incentivised advising women of the benefits of long acting reversible contraception where the explicit intent was to increase its use, and we included indicators of the uptake of long acting reversible contraception). Secondly, we required studies to have a minimum of three time points before and three after the intervention, as estimating trends is otherwise not possible.

The narrative synthesis included other studies with consistently measured data before and after incentive introduction/withdrawal but which had too few time points for interrupted time series analysis and/or which did not report (or plot) raw time series data. We excluded studies in which the population studied or quality measurement was inconsistent over time, single time point before-and-after studies without any control, studies of non-QOF financial incentives, conference abstracts, systematic and umbrella reviews, and editorials.

### Search strategy and study selection

We searched Medline, Embase, CINAHL, PsycINFO, and Scopus from 1 January 2004 to 3 September 2024, with keywords for “QOF”, “primary care”, “pay-for-performance”, and “United Kingdom” (supplementary box B details search strategies), with additional hand searching of reference lists of included studies. We imported all records into Covidence (Veritas Health Innovation, Melbourne, Australia) for deduplication, followed by title and abstract screening and full text assessment conducted independently by two reviewers. Conflicts in the screening and assessment were resolved through discussion between the two reviewers with arbitration from a third reviewer if required.

### Data extraction

We extracted key characteristics from all included studies by using a pre-developed, piloted data extraction sheet. Extracted study characteristics included publication year, region/country, data source, study intervention, study design, time frame, evaluation method, quality indicators evaluated, and relevant study data (including one and three year absolute impact if evaluated or underlying time series data if not).

Where studies reported one and three year impact, we extracted these findings from the paper and used them in analysis. One study of incentive introduction used a multilevel regression model to calculate impact.^9^ One study of incentive introduction used interrupted time series analysis and reported one and four year impact for one indicator (we included reported four year impact in the quantitative synthesis of three year impact). Two studies of incentive withdrawal used interrupted time series analysis,^4^ ^5^ one of which was a controlled interrupted time series analysis (change in Scotland where incentives were withdrawn controlled by changes in England where incentives were maintained; we include the reported controlled interrupted time series analysis results).^5^

Where studies did not report one and three year impact, we extracted raw time series data from tables where available (two studies^10^ ^11^). Where time series data were not reported in tables, we extracted raw data from time series plots by using plotdigitizer software (https://plotdigitizer.com/) (five studies^12^ ^13^ ^14^ ^15^ ^16^). This de novo reanalysis was not explicitly stated in the pre-registered protocol (https://www.crd.york.ac.uk/prospero/display_record.php?ID=CRD42023467627) as we decided on this when it became clear that multiple studies had extractable data for which de novo interrupted time series analysis would estimate impact consistent with that reported by other included studies and therefore improve coverage in the quantitative synthesis.

### De novo interrupted time series analysis methods

For each time series, we fitted models to the extracted raw data. Model choices were consistent with the original analyses, in terms of using their pre-specified timing of the intervention and whether any of the pre-intervention data points were omitted from modelling (the QOF had a “preparatory” year in 2003-04 before incentivisation when practices knew the indicators that would be used, so for some indicators performance changed beyond the previous trend in this preparatory year). We fitted interrupted time series analysis models using the *itsa* command in StataBE v18,^17^ with one and three year impact estimated using the *lincom* command.^18^ For time series with more than three pre-intervention time points, we explored whether autocorrelation was present by using the *actest* command and fitted lag terms to account for autocorrelation where needed.

For indicators with high pre-intervention performance and upward trends, predicted performance (based on previous trend) at three years could exceed 100%. In this situation, we did not estimate three year impact, but pay for performance in these circumstances cannot usually have a major impact because performance is approaching a ceiling anyway.

### Risk of bias assessment

One reviewer (LH) assessed risk of bias by using the methodological quality criteria for quantitative non-randomised studies of the Mixed Methods Assessment Tool (MMAT). A second reviewer (BG) re-scored risk of bias during data extraction but not masked to LH’s assessment, with discrepancies resolved by discussion.^19^ All of the studies included in the quantitative synthesis fulfilled all criteria for the risk of bias assessment by the MMAT (supplementary table C), meaning that they recruited representative patients/participants, conducted appropriate outcome measurements, reported outcome data completely, and accounted for confounders in data analysis.

### Quantitative synthesis

Our starting assumption was that findings should not be meta-analysed, as the diversity of indicators being incentivised meant that we did not believe that a “true” single value of pay-for-performance impact existed that could be estimated.^20^ Consistent with Cochrane Collaboration recommendations, we therefore chose to report the distribution of observed impact of incentivisation and removal of incentives for all indicators and in selected subgroups by using both quantitative synthesis (median and interquartile range of estimated impact) and graphical visualisation.^20^ We examined how impact of incentive introduction and withdrawal varied by baseline performance (value of the quality indicator in the year before the intervention) by using graphical visualisation and by estimating Pearson’s correlation coefficient. Synthesis was done separately for incentive introduction and incentive withdrawal. One paper also reported change in quality for unincentivised indicators before and after the QOF was introduced in April 2004, and we report these data as well for comparison.^21^

We categorised subgroup analysis by indicator type (simple processes such as blood pressure recording that could be done opportunistically in any consultation, complex processes such as diabetes retinal screening that could not usually be done opportunistically, intermediate outcomes such as blood pressure control, and treatments such as influenza immunisation) and by type of condition (cardiovascular disease, diabetes, and other conditions). We additionally examined impact of incentive introduction and withdrawal for 14 sets of indicators for which data were available for both. We used narrative synthesis for eligible studies in which one and three year impact was neither reported nor re-analysable.

### Patient and public involvement

The idea for the review was informed by discussions with public contributors about primary care reform in Scotland, and public contributors supported the writing of the lay summary infographic shared on social media (@acrcedincare) and policy briefing (https://doi.org/10.5281/zenodo.15372055).

## Results

The literature search after deduplication yielded 3495 records, and we screened 100 full text records for the two pre-registered research questions reported here. Thirty studies were eligible for analysis. Twenty four studies focused on introduction of incentives (including one also studying impact on non-incentivised indicators), three on withdrawal of incentives, and three on both introduction and withdrawal (supplementary figure A; supplementary tables A and B). Eleven studies were included in the quantitative synthesis. Risk of bias was low for all studies included in the quantitative synthesis (supplementary table C). Seven (35%) studies included in the narrative synthesis were judged to be at risk of bias—one because it failed to satisfy the criteria for participant representativeness as it was conducted in one particular region and six because of incomplete accounting for confounders (specifically in relation to accounting for pre-intervention trends when estimating impact).

### Quantitative synthesis of impact on measured quality

One and three year absolute percentage point change in indicators was reported or could be estimated for 83 indicators for which incentives were introduced, for 31 indicators for which incentives were withdrawn, and for 19 unincentivised indicators measured before and after introduction of the QOF in 2004 (supplementary tables D and E; supplementary figure B).

**Fig 1:**
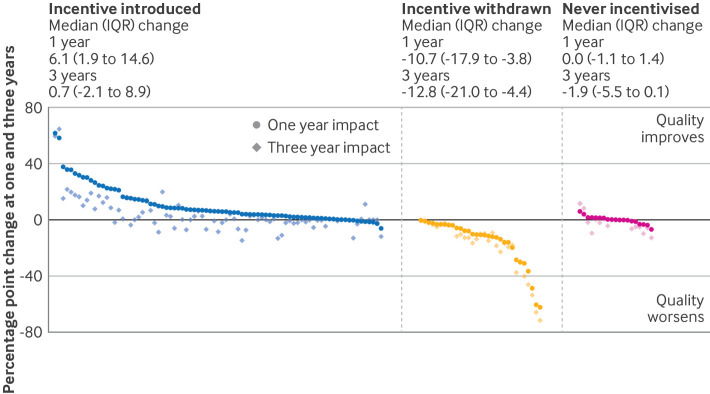
Change in recorded quality at one and three years after introduction and removal of incentives and for never incentivised indicators. Indicators ordered in descending order of one year impact/change, paired with three year impact/change on same vertical. Supplementary figures C and D show these data with indicator names. IQR=interquartile range

For all indicators, we found evidence that introduction of the incentive was usually associated with higher reported quality at one year compared with expected levels based on pre-incentivisation trends (median change 6.1 (interquartile range (IQR) 1.9 to 14.6) percentage points; 74/83 indicators improved) but not at three years (median change 0.7 (−2.1 to 8.9); 40/72 indicators improved) (table 1; fig 1; supplementary figures B-D). For incentive removal, reported quality reduced compared with expected at both one year (median change −10.7 (IQR −17.9 to −3.8)) and three years (median change −12.8 (−21.0 to −4.4). For indicators that were never incentivised, we found no consistent evidence of change from expected at one year (median change 0.0 (IQR −1.1 to 1.4)), but quality was modestly worse than expected at three years (−1.9 (−5.5 to 0.1)).

**Table 1:** Change in reported quality at one year and three years

	1 year			3 years	
	No of indicators	Median (IQR) impact or change*		No of indicators†	Median (IQR) impact or change*
**All indicators**					
Incentive introduction	83	6.1 (1.9 to 14.6)		72	0.7 (−2.1 to 8.9)
Never incentivised	19	0.0 (−1.1 to 1.4)		19	−1.9 (−5.5 to 0.1)
Incentive withdrawn	31	−10.7 (−17.9 to −3.8)		31	−12.8 (−21.0 to −4.4)
**Simple process**					
Incentive introduction	38	5.8 (2.2 to 13.8)		28	0.6 (−2.8 to 5.7)
Never incentivised	9	0.3 (−0.9 to 1.5)		9	−4.3 (−6.4 to −0.3)
Incentive withdrawn	7	−16.0 (−24.2 to −11.5)		7	−18.0 (−24.6 to −10.8)
**Complex process**					
Incentive introduction	16	24.3 (13.8 to 31.0)		15	10.1 (−2.0 to 18.3)
Never incentivised	0	-		0	-
Incentive withdrawn	7	−36.6 (−54.6 to −22.4)		7	−46.1 (−59.8 to −31.5)
**Intermediate outcome**					
Incentive introduction	14	2.6 (0.4 to 5.3)		14	−0.7 (−4.9 to 0.4)
Never incentivised	0	-		0	-
Incentive withdrawn	11	−8.0 (−10.6 to −4.7)		11	−12.7 (−15.2 to −7.6)
**Treatment and immunisation**					
Incentive introduction	15	3.8 (0.6 to 7.6)		15	3.2 (0.4 to 8.6)
Never incentivised	10	−0.1 (−2.4 to 1.0)		10	−0.2 (−4.4 to 0.2)
Incentive withdrawn	6	−3.2 (−3.7 to −1.4)		6	−2.8 (−3.4 to −1.6)
**Cardiovascular conditions**					
Incentive introduction	15	5.8 (2.6 to 11.4)		14	1.2 (−0.1 to 2.6)
Never incentivised	7	−0.01 (−2.2 to 0.8)		7	−5.0 (−7.4 to −3.2)
Incentive withdrawn	11	−10.5 (−14.2 to −6.0)		11	−13.7 (−17.4 to −7.0)
**Diabetes**					
Incentive introduction	56	6.0 (1.8 to 11.1)		46	−0.7 (−5.9 to 7.4)
Never incentivised	1	−0.3 (−0.3 to −0.3)		1	−0.3 (−0.3 to −0.3)
Incentive withdrawn	8	−4.8 (−8.7 to −3.0)		8	−7.7 (−13.2 to −3.2)
**Other conditions**					
Incentive introduction	12	13.6 (2.7 to 23.0)		12	8.7 (0.7 to 14.5)
Never incentivised	11	0.2 (−0.5 to 1.6)		11	−0.1 (−3.8 to −0.4)
Incentive withdrawn	12	−22.2 (−32.4 to −9.6)		12	−24.6 (−41.7 to −9.6)
**Paired indicators**					
Incentive introduction	16	3.8 (1.2 to 6.4)		16	1.4 (−0.9 to 4.6)
Incentive withdrawal	15	−3.9 (−9.2 to −3.2)		15	−3.9 (−11.6 to −2.2)

IQR=interquartile range.

* Positive value indicates higher observed quality than predicted by previous trend; negative value indicates lower observed quality than predicted by previous trend.

† Number is less than for one year because not all studies reported three year impact and/or de novo interrupted time series analysis could not estimate three year impact because predicted performance exceeded 100%.

**Fig 2:**
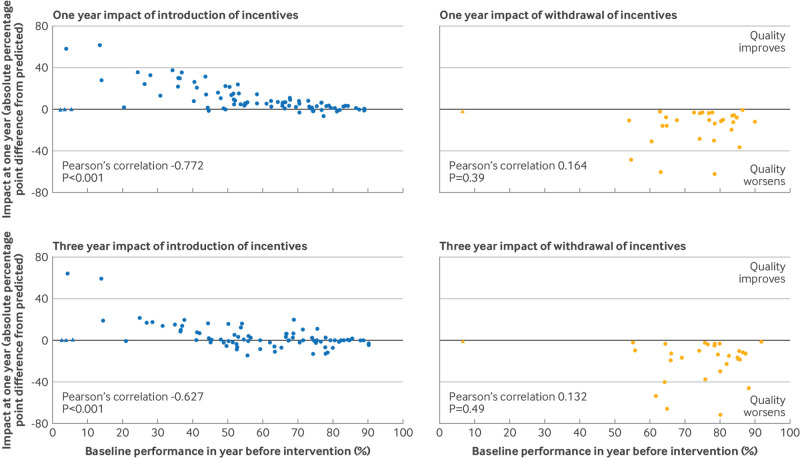
Impact of incentive introduction and withdrawal in relation to baseline performance in year before intervention. Pearson’s correlation coefficient calculated ignoring outlier points (marked with triangle), which are studies of introduction or withdrawal of incentives for long acting reversible contraception (LARC) with measures expressed as percentage of all women prescribed LARC

For incentive introduction, impact fell with increasing baseline performance in the year before introduction at both one year (Pearson r=−0.772; P<0.001) and three years (Pearson r=−0.627; P<0.001) (fig 2). For incentive withdrawal, no correlation existed between withdrawal and impact at either one year (Pearson r=0.164; P=0.39) or three years (Pearson r=0.132; P=0.49).

**Fig 3:**
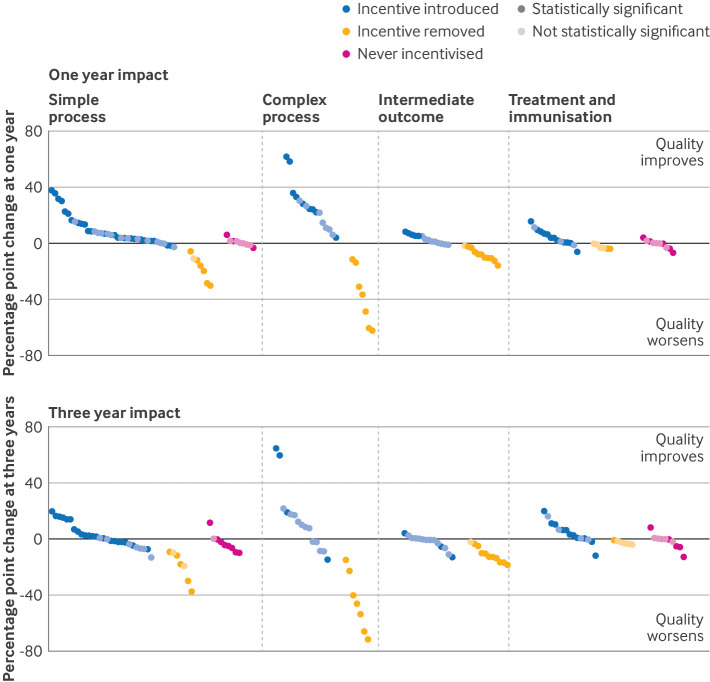
Impact of incentive introduction and incentive withdrawal at one and three years, and change in never incentivised indicators. Eleven indicators evaluable at one year are not evaluated at three years because predicted quality at three years exceeds 100%; both plots ordered in ascending order of impact at either one year or three years. Statistical significance is based on 95% confidence interval for estimate of impact not including zero, so equivalent to P<0.05

For simple process indicators (table 1; fig 3), incentive introduction was associated with improved recorded quality at one year compared with expected (median change 5.8 (IQR 2.2 to 13.8) percentage points) but not at three years (median change 0.6 (−2.8 to 5.7) percentage points). Incentive withdrawal was associated with larger reductions than expected at both one year (median change −16.0 (IQR –24.2 to −11.5)) and three years (median change −18.0 (−24.6 to −10.8)). By contrast, unincentivised simple process indicators at the time of QOF introduction in 2004 showed little change at one year but evidence of decline from expected at three years (median change −4.3 (IQR −6.4 to −0.3)).

Improvements from incentivisation of complex processes such as diabetic retinal screening were larger than those of simple processes at one year (median change 24.3 (IQR 13.8 to 31.0) percentage points) and three years (median change 10.1 (−2.0 to 18.3) percentage points). However, withdrawal of incentives for seven complex process indicators was associated with very large reductions in reported quality at three years (median change −46.1 (range −59.8 to −31.5)). Observed benefit of incentive introduction for intermediate outcomes such as blood pressure control was small and usually not statistically significant, but reported quality of intermediate outcomes worsened compared with expected when incentives were withdrawn (at three years, median change −12.7 (range −15.2 to −7.6)).

For treatment indicators, median change for incentive introduction was a small median improvement at one year (median change 3.8 (IQR 0.6 to 7.6) percentage points) and at three years (3.2 (0.4 to 8.6)), although all of the largest improvements were for five influenza immunisation indicators. For withdrawal of incentives for treatment indicators, median change was −3.2 (IQR −3.7 to −1.4) percentage points at one year and −2.8 (−3.4 to −1.6) at three years. For 10 never incentivised treatment indicators, we found little evidence of change (at three years, median change −0.2 (IQR −4.4 to 0.2)).

Patterns of change were similar to the main findings for cardiovascular and diabetes indicators but somewhat larger in magnitude for the 33 indicators relating to various other conditions (at one year, median change for incentive introduction 13.6 (IQR 2.7 to 23.0) percentage points; for incentive withdrawal −22.2 (−32.4 to −9.6) (table 1; supplementary figure E).

**Fig 4:**
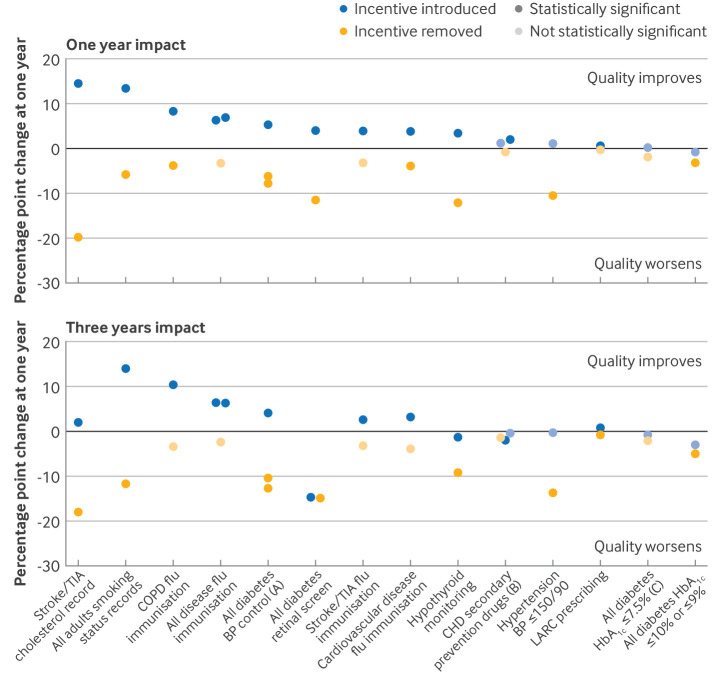
Impact of incentive introduction and withdrawal at one and three years for same/similar indicators. A: incentives introduced for blood pressure (BP) ≤145/85 mm Hg; incentive withdrawal evaluated at two levels (≤150/90 mm Hg and ≤140/80 mm Hg). B: incentives introduced for coronary heart disease (CHD) β blocker treatment and for CHD with left ventricular systolic dysfunction angiotensin converting enzyme inhibitor/angiotensin receptor blocker treatment; incentives withdrawn for CHD antithrombotic treatment. C: incentive introduction evaluated for glycated haemoglobin (HbA_1c_) ≤7.4%; incentive withdrawal evaluated for HbA_1c_ ≤59 mmol/mol (7.5%). D: incentive introduction evaluated for HbA_1c_ ≤10%; incentive withdrawal evaluated for HbA_1c_ ≤75 mmol/mol (9%). Statistical significance is based on 95% confidence interval for estimate of impact not including zero, so equivalent to P<0.05. COPD=chronic obstructive pulmonary disease; LARC=long acting reversible contraception; TIA=transient ischaemic attack

For 14 groups of indicators, data on impact was available for both incentive introduction and incentive withdrawal (table 1; fig 4; supplementary table F). We found variation in observed absolute changes but consistent evidence of improved reported quality compared with expected at one year with incentivisation (median change 3.8 (IQR 1.2 to 6.4) percentage points) and of reduced reported quality with incentive removal (median change −3.9 (−9.2 to −3.2)). Benefits of incentivisation compared with expected were smaller at three years (median 1.4 (IQR −0.9 to 4.6)), whereas median change on incentive removal was similar (−3.9 (−11.6 to −2.2)).

### Other studies of impact

A study using case note review with two time points before and two after found that in the year after implementation the QOF was not associated with any improvement in recorded quality for coronary heart disease, but a statistically significant increase occurred in recorded quality for asthma and diabetes. For all three conditions, performance did not improve further between one and three years, which the authors interpreted as being a result of ceiling effects. Continuity of care assessed by surveying patients statistically significantly worsened after QOF introduction, although absolute changes were small (supplementary table G).^9^

Other studies that could not be quantitatively synthesised found that introduction of incentives was associated with increased consultation by patients with incentivised conditions but not associated with any change in consultations for all patients in the practice.^22^ ^23^ ^24^ Processes of care were usually estimated to improve after incentivisation, but not consistently.^16^ ^25^ ^26^ ^27^ ^28^ ^29^ ^30^ Some evidence also suggested that incentivising quality in one group of people was associated with improvements in the same activity in groups in which incentives did not apply (“halo effects”) for risk factor recording,^25^ smoking cessation advice,^28^ and mean daily number of cigarettes smoked.^31^ Evidence that QOF incentives to record risk factors or control intermediate outcomes changed prescribing was mixed. For people with diabetes, blood pressure control and cholesterol control improved, but glycated haemoglobin control got worse,^32^ although other studies found evidence of earlier introduction of hypoglycaemic drugs.^33^ No evidence supported a change in prescribing in hypertension or in initiation of cardioprotective drugs in people with serious mental illness despite improved risk factor recording.^29^ ^34^ ^35^ Incentivising the use of depression severity measures at diagnosis of depression was associated with a reduction in first episodes of depression treated with antidepressants.^36^ One study of withdrawal of incentives for recording cardiovascular risk factors in people with serious mental illness found large absolute reductions in recording,^34^ whereas another found little impact on simple processes of care in multiple conditions (although the process indicators examined remained incentivised by other indicators).^37^

In relation to broader outcomes, trend adjusted QOF related ambulatory care sensitive admissions fell by 2.7% in the first year of the QOF compared with unrelated admissions and by 2.8% compared with non-ambulatory care sensitive admissions, increasing to 7.7% and 8.7% reductions respectively by year three, although the QOF was not the only relevant policy change in the period.^38^ However, UK QOF related mortality did not decline faster than expected compared with changes in a matched set of other countries.^39^

## Discussion

The introduction of QOF financial incentives was associated with one year improvements in recorded care processes compared with levels expected on the basis of pre-incentivisation trends. However, by three years performance on simple process indicators was not consistently better than expected, and improvements in complex processes were somewhat smaller than those observed at one year. Improvements in intermediate outcomes over pre-incentivisation trends were relatively small at one year and minimal at three years. Although improvements beyond trend in incentivised treatment were small, they persisted at three years. We found some evidence that improvements on introduction of incentives were potentially constrained by “ceiling effects” (whereby quality is close to a maximum before incentivisation), as the observed impact was smaller when baseline performance was higher. Withdrawal of incentives led to reductions in recorded quality of care, often matching or exceeding any gains associated with incentive introduction. We found no strong evidence of impact on unincentivised care at one year, but by three years some evidence suggested small declines in quality of unincentivised care compared with pre-incentivisation trends (potential “crowding out” of unincentivised care). One national study provided strong evidence of reductions in related ambulatory care sensitive admissions after implementation of the QOF (although causality is uncertain), but no change in QOF related mortality was seen.

### Strengths and limitations of study

Strengths of the study include systematic evaluation of the short and medium term impact of incentivising and de-incentivising multiple indicators as part of a national pay-for-performance programme with large financial incentives. The study has several limitations. Firstly, interpretation of incentive withdrawal is reasonably simple because recorded quality was stable before incentives were withdrawn,^4^ ^5^ so observed changes typically represent actual reductions from a previously steady state. By contrast, interpretation of incentive introduction is usually in the context of improving recorded quality, driven by other quality improvement work (such as national and local guideline implementation and national service frameworks) to which financial incentives were added. Compared with incentive withdrawal, therefore, a stronger assumption is that quality would have continued to improve at the same rate without financial incentives. Whether existing improvement work would have delivered the same level of improvement without incentives is not known, and at least some indicators were subject to ceiling effects that limited the scope for continued improvement. This is most obvious in evaluation of three year impact, in which predicted quality sometimes exceeded 100% (in this situation, we chose not to estimate three year impact of incentive introduction as the counterfactual is implausible). Ceiling effects potentially mean that the impact of incentive introduction is being underestimated, although patterns are consistent even for indicators for which recorded quality is some distance from 100% (supplementary figure B).

Secondly, evaluation of intermediate outcome indicators is problematic because under the QOF, failure to record an intermediate outcome is assumed to mean that the outcome target is not achieved. This means that changes in the process indicator may lead to changes in the intermediate outcome indicator even if actual (but unrecorded) intermediate outcomes are the same. Caution is therefore needed when interpreting impact for intermediate outcome indicators. Similar considerations apply to some complex processes that were not routinely recorded before incentivisation (such as pre-conceptual advice in women with epilepsy). As this care was not routinely recorded historically, no evaluations of incentive introduction are available, but incentive withdrawal shows very large reductions in reported quality that may just represent a return to not routinely recording this activity. Caution is therefore needed in directly comparing the impact of introduction and withdrawal because the indicators being evaluated are different. However, our paired indicator analysis is consistent with the overall findings.

Thirdly, although related ambulatory care sensitive admissions fell after implementation of the QOF, this was not the only improvement programme in the UK that covered these, so interpretation of this finding should be cautious. These considerations highlight that “recorded quality” can have a variable relation with actual delivery of care. Finally, the focus of this analysis has been incentivised indicators, which risks ignoring harms of pay-for-performance programmes. This review found some evidence of “crowding out” of unincentivised care for 19 indicators reported by one study,^21^ and that patients’ perceptions of continuity worsened.^9^ Consistent with this, multiple qualitative studies have found that many general practice staff perceived that the focus on QOF indicators led to less attention being paid to both unincentivised aspects of care and to patients’ concerns, with perceived risk of reductions in personal continuity.^40^ Given QOF implementation in a resource constrained system, tunnel vision and crowding out are likely to have occurred more widely and are likely in any substantial pay-for-performance programme.^41^

### Comparison with other studies

The findings are consistent with previous international narrative reviews which concluded that introduction of pay for performance has inconsistent benefit, with improvements largely observed in the short term for processes of care and larger impact when baseline performance was lower.^1^ ^2^ Incentive withdrawal is less well studied outside of the QOF, but two randomised trials of hypertension care in the US and chlamydia screening in Australia also found that improvements following incentivisation were not sustained when incentives were withdrawn,^6^ ^42^ which was also observed in a UK non-QOF programme of incentivisation of screening and brief intervention for alcohol misuse.^43^ The QOF used financial incentives that were larger than in most other countries,^2^ implemented indicators that usually had wide professional acceptance (although baseline performance was therefore often already high), aligned incentives to reputational incentives through public reporting,^44^ and provided implementation and educational support.^45^ ^46^ Practices seemed to respond with several organisational changes,^40^ ^47^ ^48^ ^49^ but despite initial gains in recorded quality this study is not consistent with the QOF driving sustained quality improvement over and above existing trends. Notably, performance on many indicators was already improving before incentivisation (because of other quality improvement initiatives such as professional education and national clinical guidelines, with consequent increasing implementation of registers, recall systems, and structured review of people with chronic conditions); and, for some indicators with high baseline performance, impact was likely subject to ceiling effects. Any improvements seen with QOF incentivisation were also not sustained in the sense that withdrawal of incentives was associated with reductions in recorded quality. This may have been because the external financial incentives of the QOF reduced internal motivation to maintain quality; it suggests that reorganisation to deliver QOF reporting requirements may not have been optimised to deliver sustainable quality improvement and may have overshadowed other improvement activity.^40^

Some of the gains in recorded quality with incentive introduction (and losses after withdrawal) probably simply reflect changes in recording and in some cases indicator gaming (an example of which is that blood pressure recordings which contributed to QOF payment were systematically lower, and at the payment target, compared with blood pressure recordings at other times of the year which did not contribute to payment).^50^

### Implications

The findings of this study emphasise that pay for performance is no magic bullet to improve quality. Pay-for-performance programme designers face the challenge of knowing whether their programme delivers meaningful improvement and whether incentives are driving organisational change that will sustain improvement when incentives are withdrawn. Deployment of pay for performance therefore needs to evaluate the impact of incentive introduction and withdrawal, ideally by independent measurement rather than relying on data reported for payment. More broadly, whether or not the QOF “worked” is a complicated question, because it was never purely a quality improvement initiative. The original bargain between government and general practitioners was that general practices would deliver higher quality of care and in return would receive increased income (which both sides agreed was needed to tackle low morale and problems of recruitment and retention of general practitioners).^45^ ^46^ Both sides of this bargain were delivered, but over time government came to believe that it was locked into paying for steady state (rather than improving) quality, whereas general practitioners saw steady declines in practice income after adjustment for inflation, with falling morale, increasing intention to leave practice, and resurgent problems of recruitment and retention.^51^

More broadly, in the 20 years since the QOF was implemented, the challenges posed to health systems by population ageing and the rising prevalence of multiple long term conditions and frailty have been increasingly recognised. The QOF remains one of the largest ever attempts to improve healthcare quality by using pay for performance, using the approach of deploying financial incentives to improve narrow, disease focused indicators. Given that the QOF did not seem to drive sustained change in these narrow indicators after incentives were withdrawn, an unmodified QOF approach is unlikely to be useful for increasing the delivery of very complex behaviours such as care coordination and management of complex problems such as polypharmacy.^52^ ^53^ However, pay for performance or other kinds of financial incentive might have a role to play if deployment aligns them with other improvement mechanisms such as outreach professional education,^54^ audit and feedback,^55^ or collaborative improvement models,^56^ which may have more persistent effects on quality of care.^42^ Notably, more recent QOF incentives in England include payment for participation in national quality improvement programmes rather than delivery of narrow, disease focused indicators.^57^ Such approaches may be better at engaging and sustaining professionals’ internal motivation to improve care than narrow pay-for-performance programmes,^58^ although this requires further evaluation.

### Conclusion

The UK primary care pay-for-performance programme initially improved quality of care as measured by the indicators examined in this study beyond that predicted by pre-incentivisation trends, but improvements associated with incentivisation were not persistent and seemed to reverse after withdrawal of incentives.

## What is already known on this topic

The UK Quality and Outcomes Framework (QOF) is one of the largest and longest running healthcare pay-for-performance programmes in the world
Pay for performance in healthcare has been deployed in many countries, but its effectiveness is still uncertain
Differences in context make comparing pay-for-performance programmes difficult

## What this study adds

Introduction of QOF incentives was associated with an immediate improvement in recorded quality at one year compared with previous trends but not by three years
Withdrawal of incentives was associated with reductions in recorded quality at both one and three years, which were similar in size to the gains observed with incentive introduction
Whether or how best to deploy financial incentives in primary care therefore remains uncertain

## Data Availability

Extracted raw data used in interrupted time series analysis (ITSA) modelling and Stata code used in ITSA analysis are available from https://doi.org/10.5281/zenodo.14951135.

## Web extra

Extra material supplied by authors

Web appendix: Supplementary materials

## Notes

Contributors: BG and SWM conceived the study. LH led literature searching, title/abstract and full text screening (to which all authors contributed), and data extraction. BG did the interrupted time series (re)analysis. All authors contributed to interpretation of the findings. BG and LH initially drafted the paper, with all authors reviewing drafts, contributing to the writing, and approving the final version. BG is the guarantor. The corresponding author attests that all listed authors meet authorship criteria and that no others meeting the criteria have been omitted.

Transparency: The lead author (the manuscript’s guarantor) affirms that this manuscript is an honest, accurate, and transparent account of the study being reported; that no important aspects of the study have been omitted; and that any discrepancies from the study as planned (and, if relevant, registered) have been explained.

Dissemination to patient and public communities: Public contributors supported the writing of the lay summary shared on social media (@acrcedincare) and policy briefing (https://doi.org/10.5281/zenodo.15372055).

Provenance and peer review: Not commissioned; externally peer reviewed
