# Supplementary material for: Effect of UK Quality and Outcomes Framework pay-for-performance programme on quality of primary care: systematic review with quantitative synthesis
Source: BMJ. 2025 Jun 25;389:e083424. doi: 10.1136/bmj-2024-083424 (PMC12188983; doi:10.1136/bmj-2024-083424)
Supplement: Supplementary file 1 — Web appendix: Supplementary materials [file hole083424.ww1.pdf]

Effect of UK Quality and Outcomes Framework pay-for-performance programme on quality of primary care: systematic review with quantitative synthesis

Authors

Ho L, Mercer SW, Henderson D, Donaghy E, Guthrie B.

Effect of UK Quality and Outcomes Framework pay-for-performance programme on quality of primary care: systematic review with quantitative synthesis 1

Supplementary Box A: Brief description of the UK Quality and Outcomes Framework[1-3] 2

Supplementary Box B: Search strategies for databases 4

Supplementary Figure A: PRISMA Flow Diagram 6

Supplementary Figure B: Interrupted time-series figures for indicators where new models were fitted (all incentive introduction except Pasvol 2022 which is both introduction and withdrawal). 7

Supplementary Figure C: Impact at one and three years for incentive introduction 13

Supplementary Figure D: Impact at one and three years for incentive withdrawal and never incentivised 14

Supplementary Figure E: Impact at one and three years by condition group 15

Supplementary Table A. Characteristics of included studies 16

Supplementary Table B: Studies excluded after full-text review 22

Supplementary Table C. Risk of bias assessment of included studies 26

Supplementary Table D: Interrupted time-series analysis models for quality of care indicators where new modelling done 28

Supplementary Table E: Impact at one year and three years on quality of care indicators (difference between predicted based on prior trend, and estimated from model given step-change and change in trend) 31

Supplementary Table F: Matched indicators with one and three year impact data for both incentive introduction and incentive withdrawal 36

Supplementary Table G: Summary of findings of studies included in the narrative synthesis 37

Supplementary Box A: Brief description of the UK Quality and Outcomes Framework[1-3]

Wider context

The UK has a state-funded National Health Service (NHS) which is free at the point of care (with the exception of fairly small co-payments for some community prescriptions and some dental services). Patients register with a single general practice which provides all primary medical care (including doing all community prescribing) and is a gatekeeper for almost all specialist care (the main exception being emergency care where patients can self-refer to the emergency department). General practices historically all physician-owned businesses which contracted to provide NHS care. In the early 2000s, there was a major crisis of recruitment and retention in general practice, significantly driven by falling practice incomes and rising workload. The broad shape of the deal that became the 2004 new General Medical Services (nGMS) contract was a renegotiation of funding with three core elements: (1) A revised capitation formula (~75% of income); (2) The Quality and Outcomes Framework (QOF) pay-for-performance programme (~20%); (3) Flexibly commissioned 'enhanced services' contracts for national and local priorities not covered by capitation (~5%). nGMS implementation also included other changes such as significant investment in information technology and support to make better use of electronic health records, and public reporting of QOF performance (ie non-financial incentives for QOF quality). A key element of the nGMS 'deal' was that practices got a substantial increase in outcome in return for improved quality of care, because new funding was largely via QOF payments.

Quality and Outcomes Framework 2004-2013

In this period, QOF was a UK wide system (whereas most other elements of NHS care were devolved to country level [England, Scotland, Wales, Northern Ireland] with some divergence in organisation). The initially incentivised ~150 indicators were a mix of:

1. Structure (eg providing a minimum appointment length, maintaining disease registers, running an approved annual patient experience survey)
2. Process (eg simple process measures like BP recording and more complex process measures like retinopathy screening in diabetes, all to be done within a defined period)
3. Intermediate outcome (eg blood pressure control in hypertension) where 'not recently recorded' meant that the outcome indicator was failed.
4. Treatment indicators (eg antithrombotic therapy in coronary heart disease)

Incentives were paid on a sliding scale depending on performance, with no money paid until performance crossed a lower threshold, and payment increasing linearly up to a maximum threshold. Payment was non-competitive and there were no penalties. Practices were also allowed to exception report patients who refused three offers of review, or who were unsuitable for the indicator (eg because terminally ill, or allergic to/intolerant of the incentivised drug).

Over the period 2004-2013, the structural indicators were largely removed with the exception of maintaining disease registers, some of the process indicators were retired as performance was near maximum (although many processes remained incentivised by linked intermediate outcome indicators), and a number of new disease domains were added (some of which were controversial eg indicators to use instruments like PHQ9 or HADS at diagnosis of depression were widely disliked by clinicians, and drove a number of unintended consequences such as changes in how mood disorders were coded).

Quality and Outcomes Framework 2013 onwards

Both sides of the QOF bargain ended up dissatisfied with it. From a GP perspective, income has declined since QOF implementation despite rising workload, and recruitment and retention is again a major problem. However, practices still had to deliver multiple QOF indicators. From an NHS and government

perspective, the quality gains from QOF were less than expected and the initial cost higher than expected (primarily because performance before QOF was already much better than they believed at the time), and quality rapidly hit a ceiling. Given QOF's initial very broad scope, it also proved difficult to identify new indicators which were robust enough to incentivise, with both sides unhappy - GPs because new indicators were not always clinically convincing; NHS/government because new indicators were sometimes tick box raising suspicions that care did not necessarily improve (for example, providing pre-conception advice to women taking antiepileptics is clearly important, but it is hard to specify an indicator definition that is not a tick box).

Although there was increasing divergence between UK countries from 2013, the general move was towards a reduction in the number of indicators and the proportion of practice income tied to QOF, although with variation between countries. Scotland abolished QOF in 2016 as part of new GP contract negotiations, and the other UK countries have scaled it back to varying degrees, with NHS England consulting with stakeholders in 2023/2024 on whether QOF should be continued, and if so, then where and how should incentives be deployed.

* PHQ9: Patient Health Questionnaire 9 item; HADS: Hospital Anxiety and Depression Scale

Supplementary Box B: Search strategies for databases

Medline via Ovid:
1. "QOF".mp.
2. "Quality and Outcomes Framework".mp.
3. "Quality Outcomes Framework".mp.
4. 1 or 2 or 3
5. "Financial incentive$".mp.
6. "pay-for-performance".mp.
7. 5 or 6
8. "Primary care".mp.
9. ("General practi$" or "family medicine" or "family doctor$").mp.
10. 8 or 9
11. (United Kingdom or UK or England or Scotland or Wales or Northern Ireland).mp.
12. 7 and 10 and 11
13. 4 or 12
14. 7 and 10
15. 4 or 14

Embase via Ovid:
1. "QOF".mp.
2. "Quality and Outcomes Framework".mp.
3. "Quality Outcomes Framework".mp.
4. 1 or 2 or 3
5. "Financial incentive$".mp.
6. "pay-for-performance".mp.
7. 5 or 6
8. "Primary care".mp.
9. ("General practi$" or "family medicine" or "family doctor$").mp.
10. 8 or 9
11. (United Kingdom or UK or England or Scotland or Wales or Northern Ireland).mp.
12. 7 and 10 and 11
13. 4 or 12
14. 7 and 10
15. 4 or 14

PsycINFO via Ovid:
1. "QOF".mp.
2. "Quality and Outcomes Framework".mp.
3. "Quality Outcomes Framework".mp.
4. 1 or 2 or 3
5. "Financial incentive$".mp.
6. "pay-for-performance".mp.
7. 5 or 6
8. "Primary care".mp.
9. ("General practi$" or "family medicine" or "family doctor$").mp.
10. 8 or 9
11. (United Kingdom or UK or England or Scotland or Wales or Northern Ireland).mp.
12. 7 and 10 and 11
13. 4 or 12
14. 7 and 10
15. 4 or 14

CINAHL:

S1. TX ("QOF" OR "Quality and Outcomes Framework" OR "Quality Outcomes Framework")
S2. TX ("Financial incentive" OR "Financial incentives" OR "Pay-for-performance")
S3. TX ("Primary care" OR "General practice" OR "General practices" OR "Family medicine" OR "Family doctor" OR "Family doctors")
S4. S2 AND S3
S5. S1 OR S4

Scopus:
(((TITLE-ABS-KEY ("Primary care") OR TITLE-ABS-KEY ("Family medicine") OR TITLE-ABS-KEY ("Family doctor") OR TITLE-ABS-KEY ("Family doctors") OR TITLE-ABS-KEY ("General practice") OR TITLE-ABS-KEY ("General practices"))) AND ((TITLE-ABS-KEY ("Financial incentive") OR TITLE-ABS-KEY ("Financial incentives") OR TITLE-ABS-KEY ("Pay-for-performance")))) OR ((TITLE-ABS-KEY (qof) OR TITLE-ABS-KEY ("Quality and Outcomes Framework") OR TITLE-ABS-KEY ("Quality Outcomes Framework")))

Supplementary Figure A: PRISMA Flow Diagram

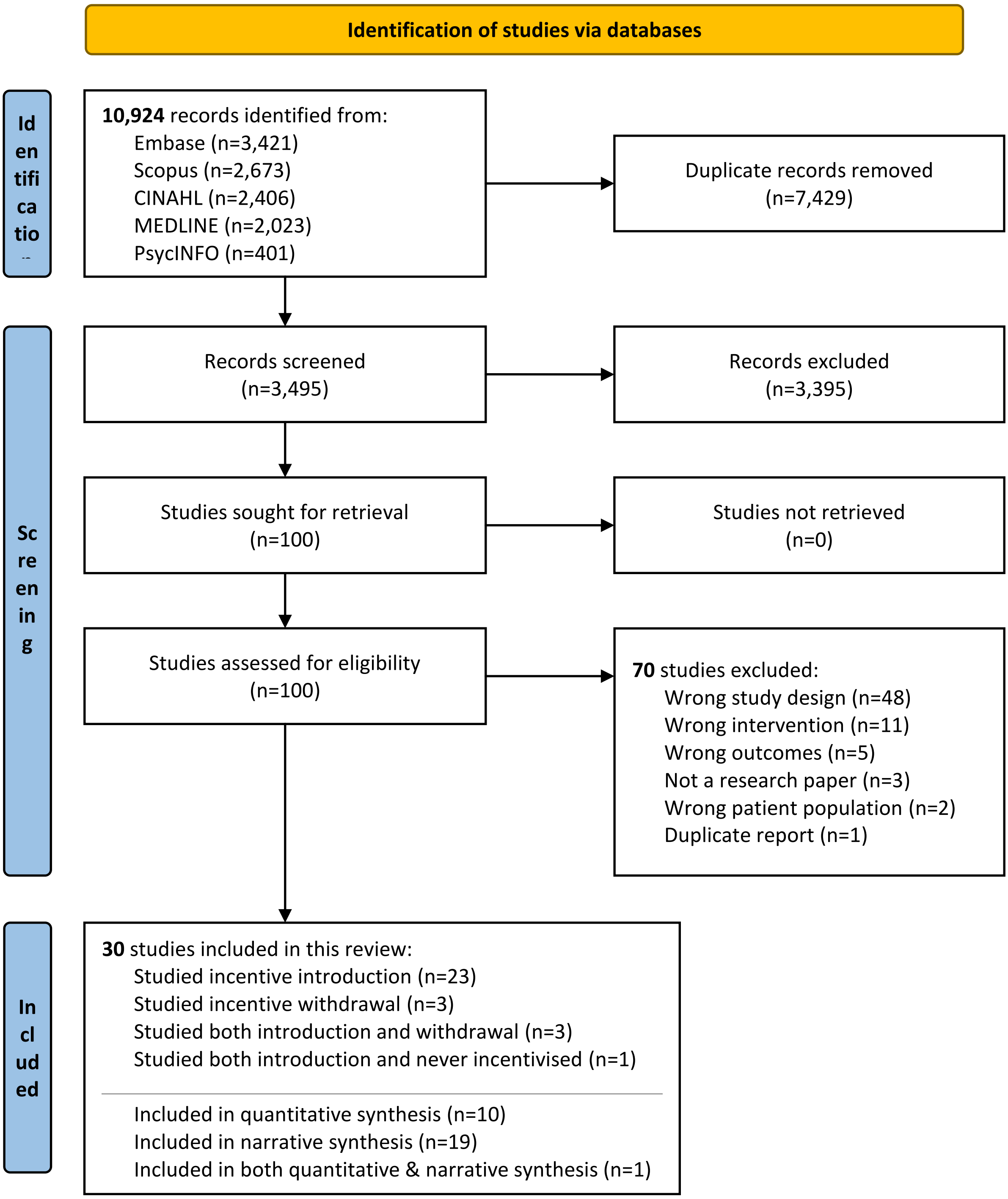

Supplementary Figure B: Interrupted time-series figures for indicators where new models were fitted (all incentive introduction except Pasvol 2022 which is both introduction and withdrawal).

Arrowsmith 2014 LARC prescribing rate

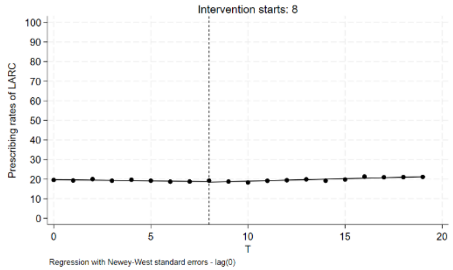

Calvert 2009 Type 1 diabetes BMI record

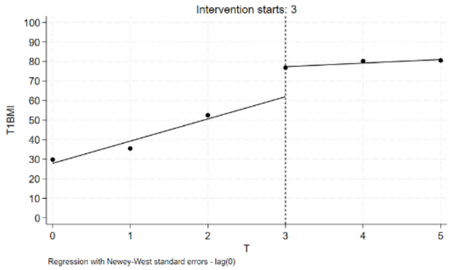

Calvert 2009 Type 1 diabetes smoking status record

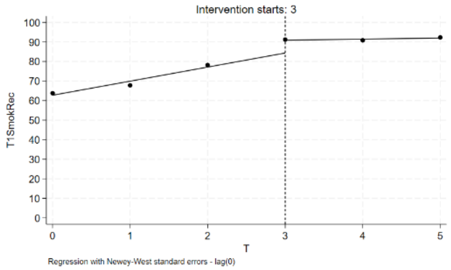

Calvert 2009 Type 1 diabetes HbA1c record

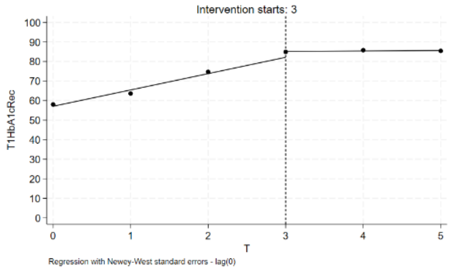

Calvert 2009 Type 1 diabetes BP record

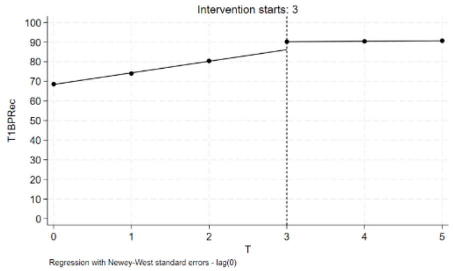

Calvert 2009 Type 1 diabetes eGFR record

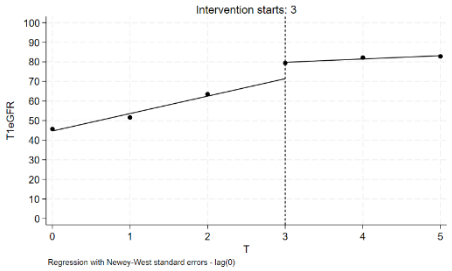

Calvert 2009 Type 1 diabetes cholesterol record

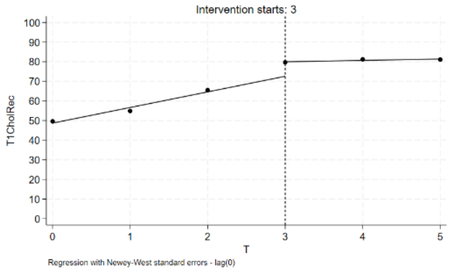

Calvert 2009 Type 1 diabetes smoking cessation advice record

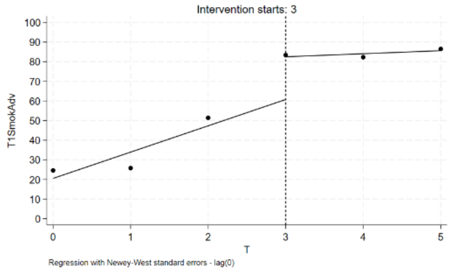

Calvert 2009 Type 1 diabetes retinopathy screening record

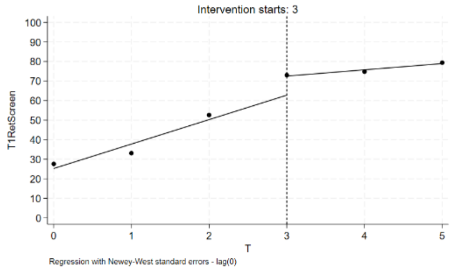

Calvert 2009 Type 1 diabetes peripheral pulses record

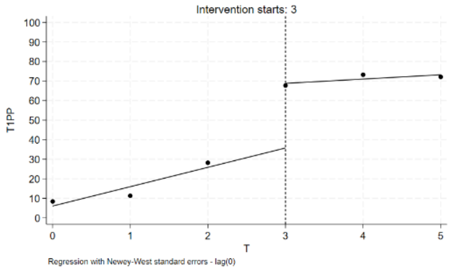

Calvert 2009 Type 1 diabetes neuropathy test record

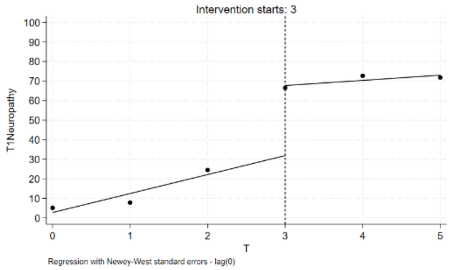

Calvert 2009 Type 1 diabetes microalbuminuria record

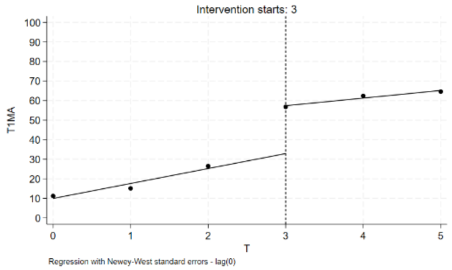

Calvert 2009 Type 1 diabetes cholesterol ≤5mmol/l

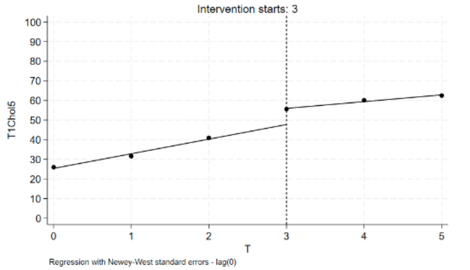

Calvert 2009 Type 1 diabetes HbA1c≤7.5%

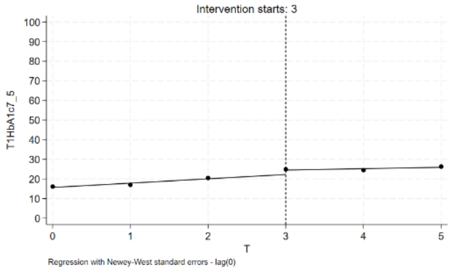

Calvert 2009 Type 1 diabetes HbA1c≤10%

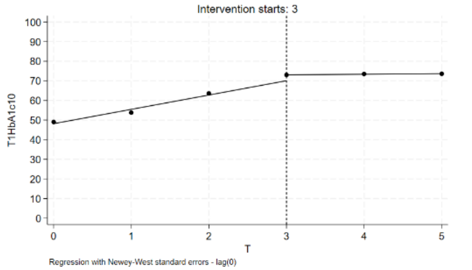

Calvert 2009 Type 1 diabetes BP≤145/85mmHg

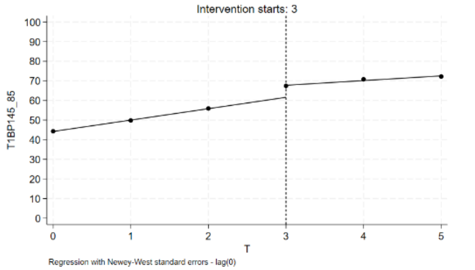

Calvert 2009 Type 1 diabetes ACE/ARB for microalbuminuria

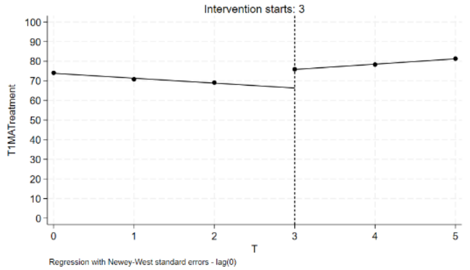

Calvert 2009 Type 1 diabetes flu immunisation record

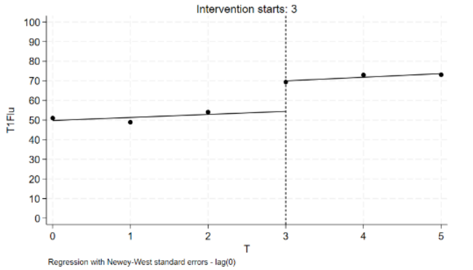

Calvert 2009 Type 2 diabetes BMI record

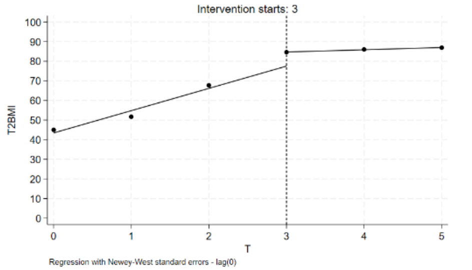

Calvert 2009 Type 2 diabetes smoking status record

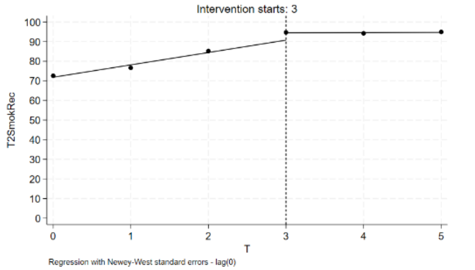

Calvert 2009 Type 2 diabetes HbA1c record

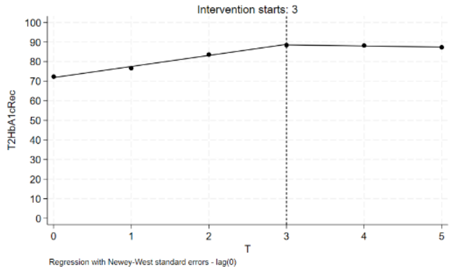

Calvert 2009 Type 2 diabetes BP record

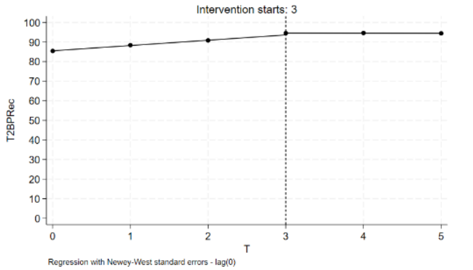

Calvert 2009 Type 2 diabetes eGFR record

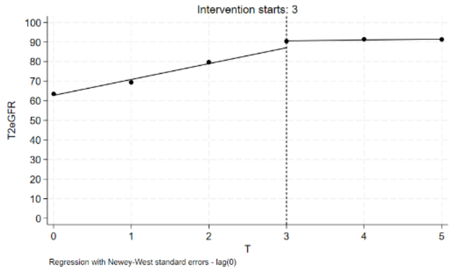

Calvert 2009 Type 2 diabetes cholesterol record

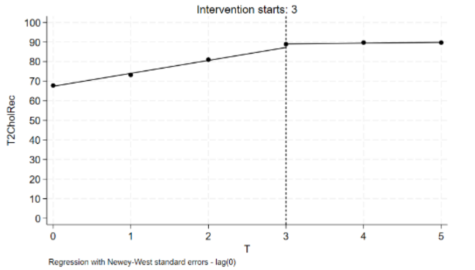

Calvert 2009 Type 2 diabetes smoking cessation advice record

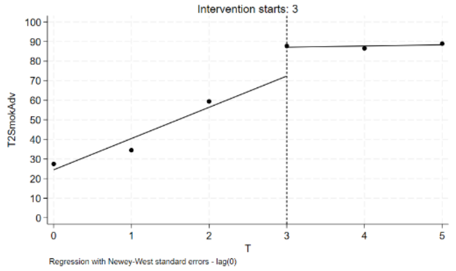

Calvert 2009 Type 2 diabetes retinopathy screening record

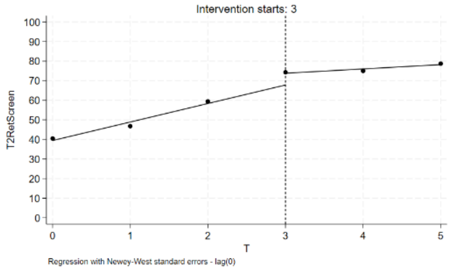

Calvert 2009 Type 2 diabetes peripheral pulses record

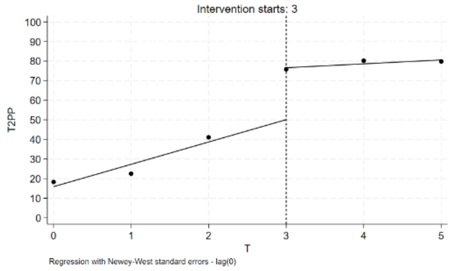

Calvert 2009 Type 2 diabetes neuropathy test record

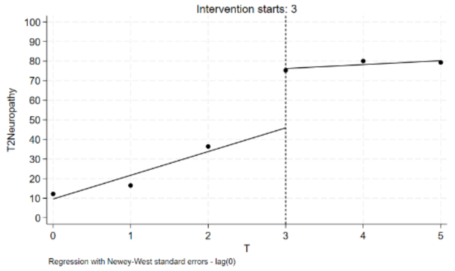

Calvert 2009 Type 2 diabetes microalbuminuria record

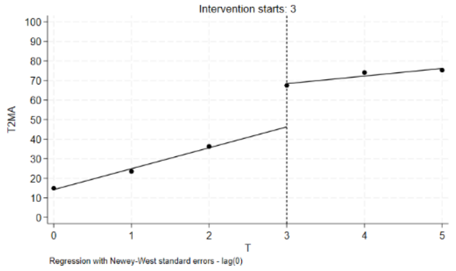

Calvert 2009 Type 2 diabetes cholesterol ≤5mmol/l

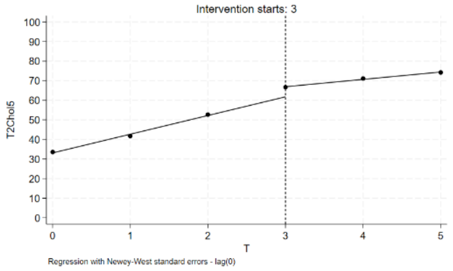

Calvert 2009 Type 2 diabetes HbA1c≤7.5%

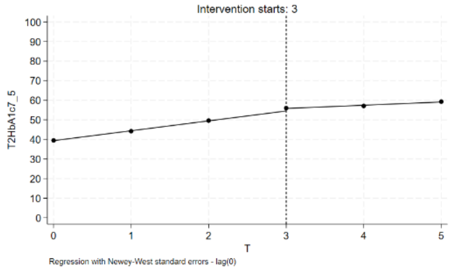

Calvert 2009 Type 2 diabetes HbA1c≤10%

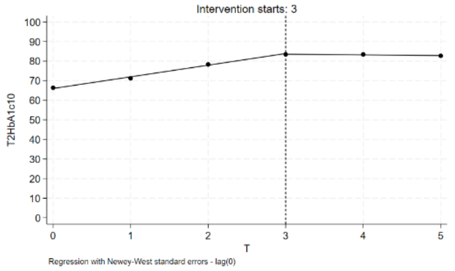

Calvert 2009 Type 2 diabetes BP≤145/85mmHg

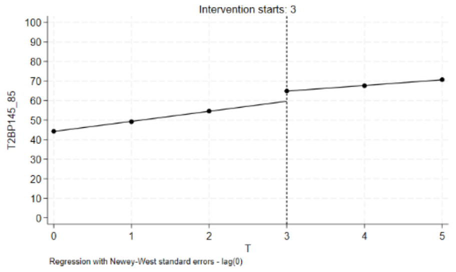

Calvert 2009 Type 2 diabetes ACE/ARB for microalbuminuria

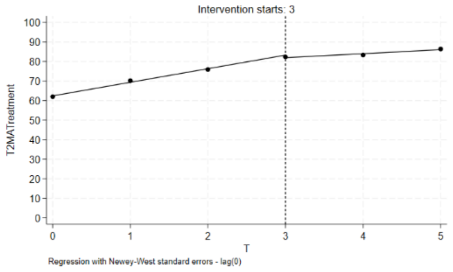

Calvert 2009 Type 2 diabetes flu immunisation record

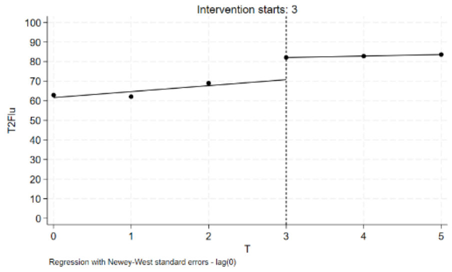

Kontopantelis 2013 All diabetes BMI record

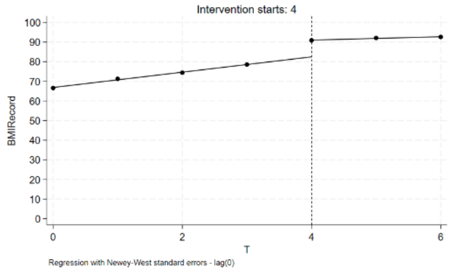

Kontopantelis 2013 All diabetes smoking record

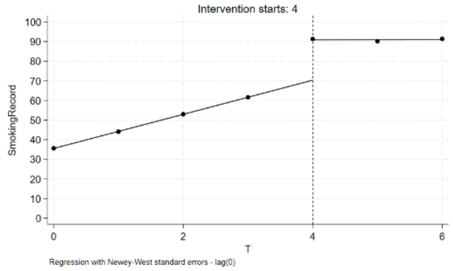

Kontopantelis 2013 All diabetes HbA1c record

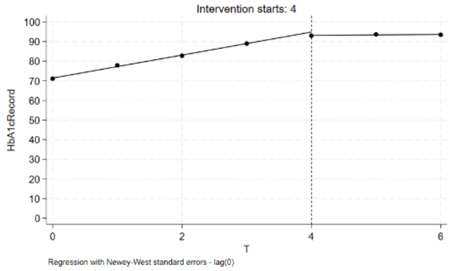

Kontopantelis 2013 All diabetes blood pressure record

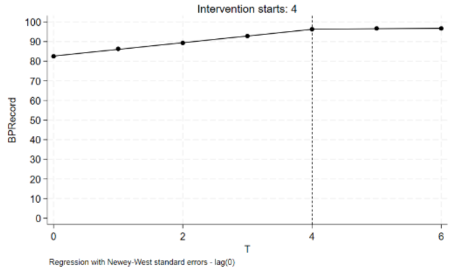

Kontopantelis 2013 All diabetes creatinine record

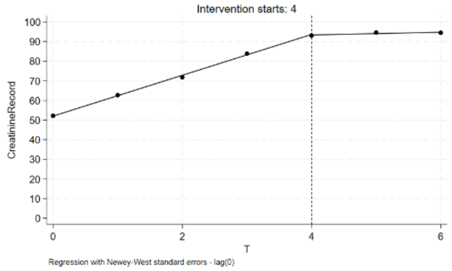

Kontopantelis 2013 All diabetes cholesterol record

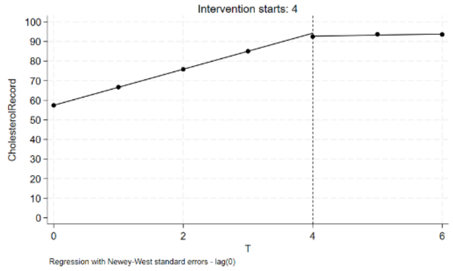

Kontopantelis 2013 All diabetes smoking advice record

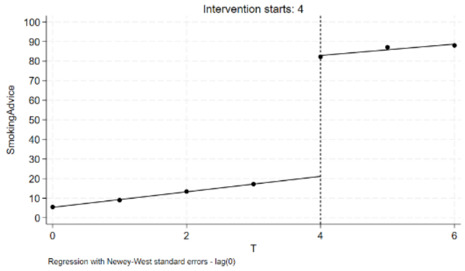

Kontopantelis 2013 All diabetes retinopathy screening record

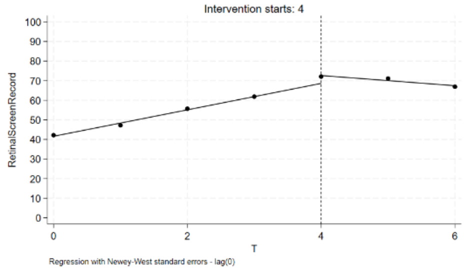

Kontopantelis All diabetes peripheral pulses record

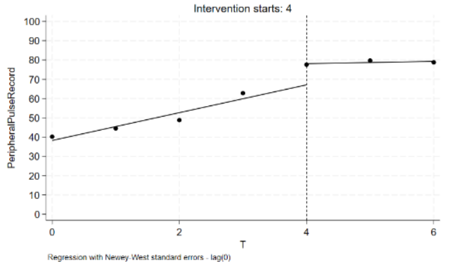

Kontopantelis 2013 All diabetes neuropathy test record

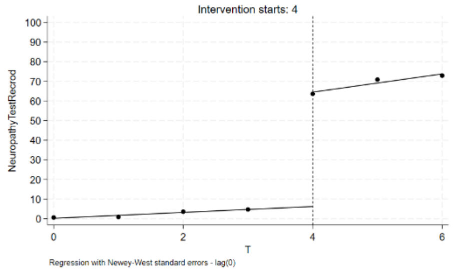

Kontopantelis 2013 All diabetes microalbuminuria record

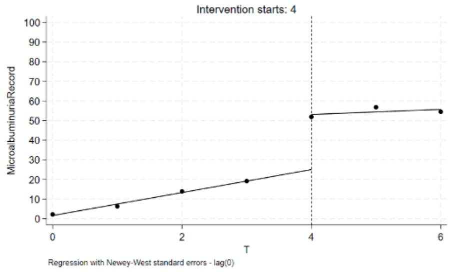

Kontopantelis 2013 All diabetes HbA1c ≤7.4%

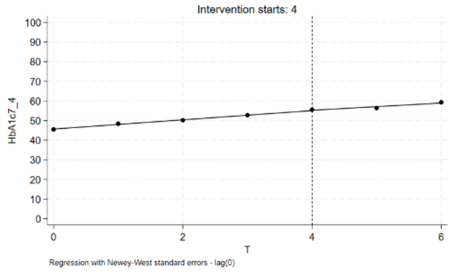

Kontopantelis 2013 All diabetes HbA1c ≤10%

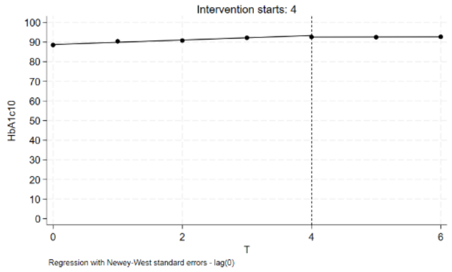

Kontopantelis 2013 All diabetes BP≤145/85mmHg

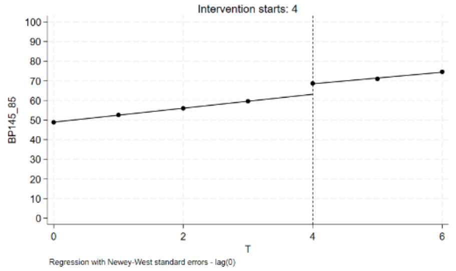

Kontopantelis 2013 All diabetes cholesterol <5mmol/l

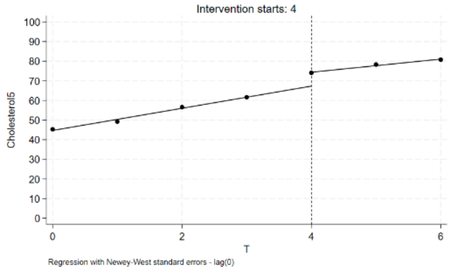

Kontopantelis 2013 All diabetes ACEI/ARB treatment in microalbuminuria

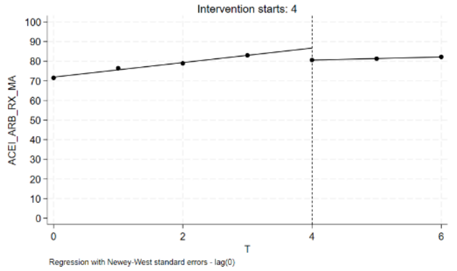

Kontopantelis 2013 flu immunisation record

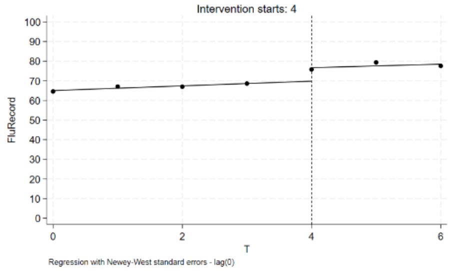

Matias 2024 Serious mental illness BMI record

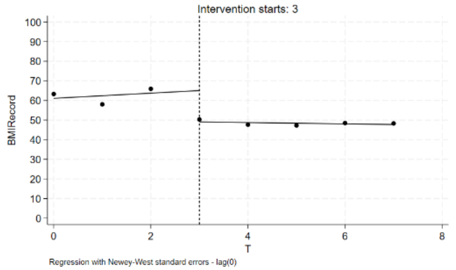

Matias 2024 Serious mental illness cholesterol record

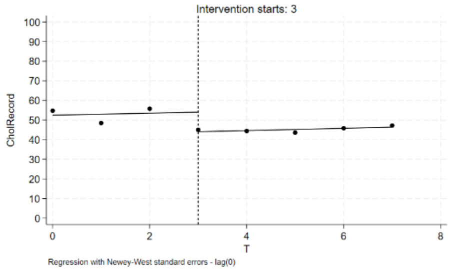

Pasvol 2022 LARC prescribing rate (introduction is first intervention)

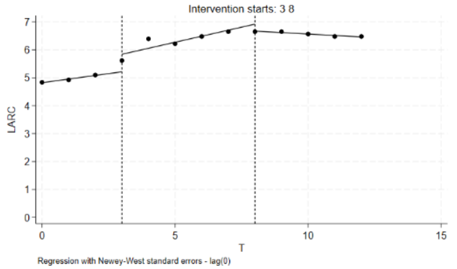

Pasvol 2022 LARC prescribing rate (withdrawal is second intervention)

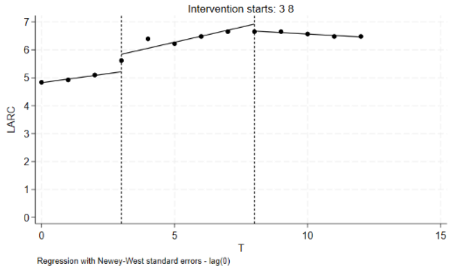

Simpson 2011 Hypertension BP record

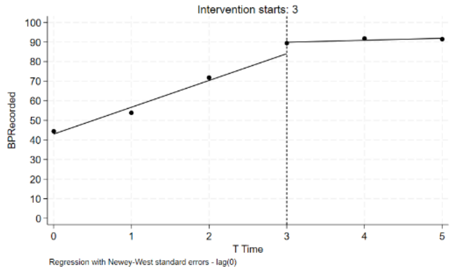

Simpson 2011 Hypertension BP≤140/90mmHg

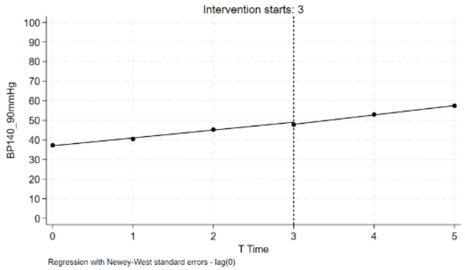

Simpson 2011 Hypertension BP≤150/90mmHg

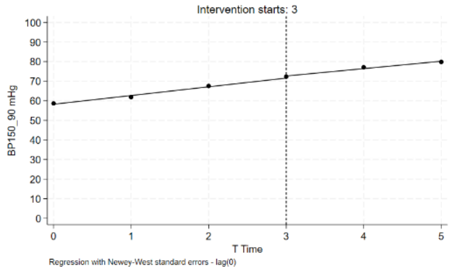

Taggar 2012 All patients smoking status record

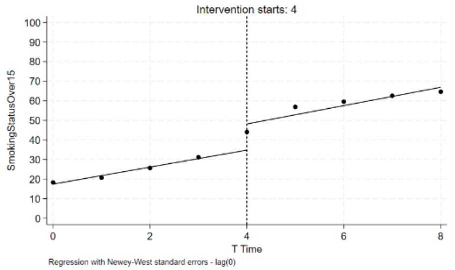

Taggar 2012 – Chronic condition smoking status record	Taggar 2012 – Chronic condition smoking cessation advice record
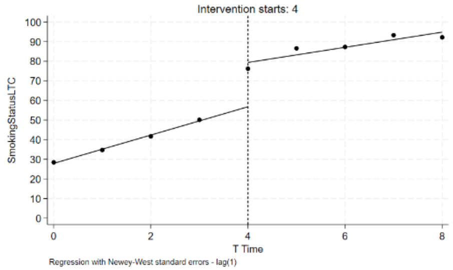	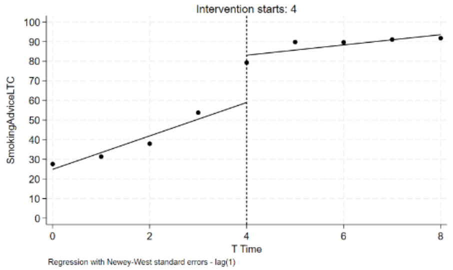

Supplementary Figure C: Impact at one and three years for incentive introduction

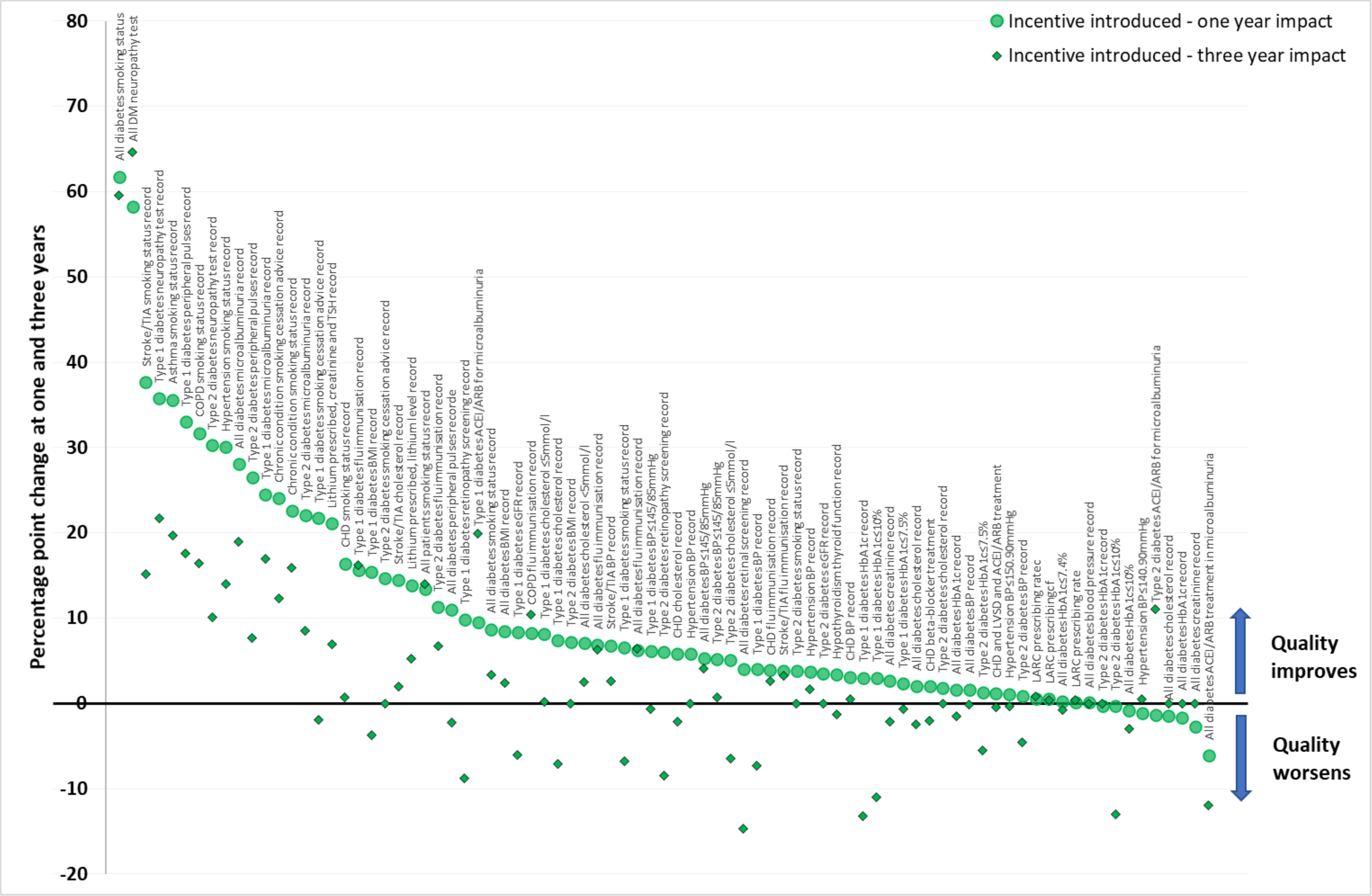

Supplementary Figure D: Impact at one and three years for incentive withdrawal and never incentivised

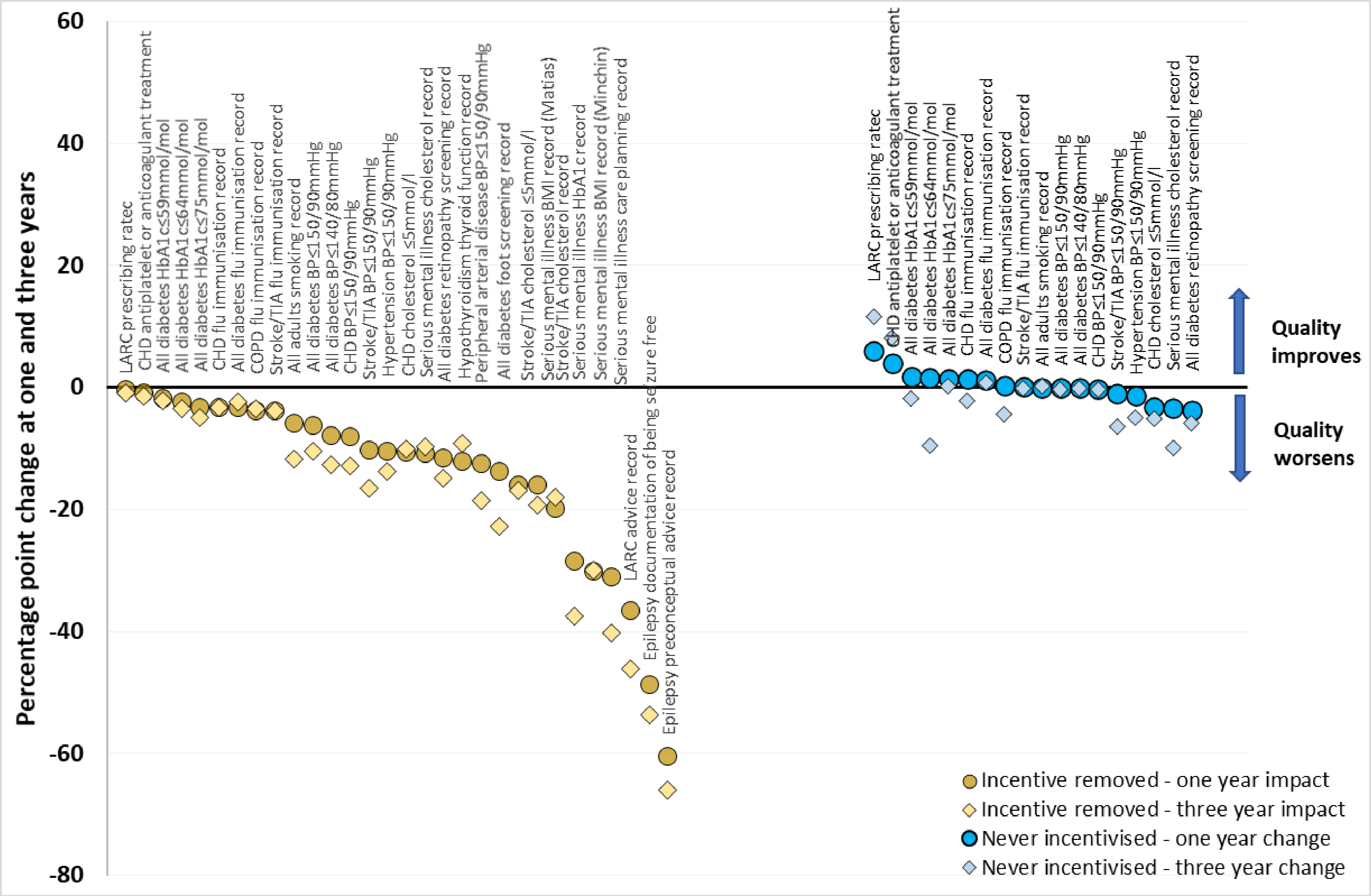

Supplementary Figure E: Impact at one and three years by condition group

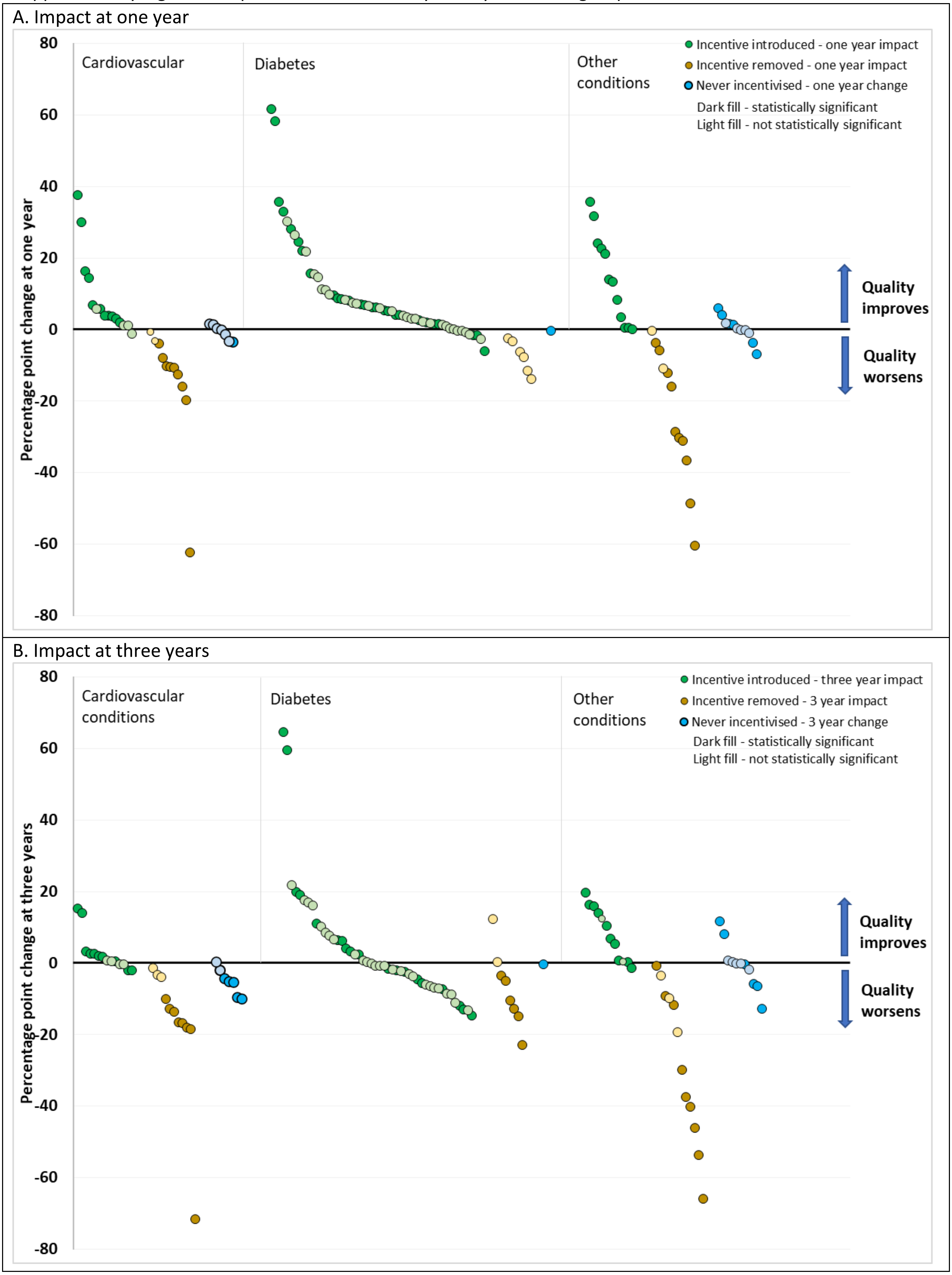

11 indicators evaluable at one-year are not evaluated at three years because predicted quality at three years exceeds 100%; both plots ordered in descending order or impact at either one year or three years. Statistical significance is based on the 95% confidence interval for the estimate of impact not including zero, so equivalent to $p<0.05$.

Supplementary Table A. Characteristics of included studies

Author, year (Region/Country)	Data source	Intervention type (detail)	Study design (Time frame)	No. of practices/ List or simple size	Inclusion criteria for study population	Evaluation method	Synthesis method
Arrowsmith 2014 (England)[4]	PACT data from the NHS England Prescription Pricing Authority	**Incentive introduction** (QOF indicator for long-acting reversible contraception advice in 2009)	ITSA (April 2007 to March 2012)	581 practices<br>Mean list size of 1577 (range 54 to 8868)	Whole population of England	Based on quarterly data, to examine long-acting reversible contraception prescribing patterns in primary care before and after the introduction of QOF contraception indicators in 2009.	Quantitative synthesis<br>New ITSA model fitted from data extracted from plot in original paper
Calvert, 2009 (UK)[5]	Doctors' Independent Network Database	**Incentive introduction** (QOF diabetes indicators in 2004)	Retrospective cohort (April 2002 to March 2007)	147 practices<br>Mean list size of 8929 (SD 4147)	Patients with a Read code for diabetes or one or more prescriptions for oral antidiabetic drugs, insulin, or glucose testing kits	Based on annual data, to examine the management of diabetes between 2001 and 2007 and assess whether changes in the quality of care reflect existing temporal trends or are a direct result of the implementation of QOF.	Quantitative synthesis<br>New ITSA model fitted from data extracted from table in original paper
Campbell, 2009 (England)[6]	Case note review	**Incentive introduction** (multiple QOF indicators in 2004	Case note review 1998 ,2003, 2005 and 2007	42 general practices	General practices recruited to be representative of England, patients defined by multiple quality indicators	"Interrupted time-series analysis" although only two timepoints before and two after intervention	Narrative synthesis (not enough data points for ITSA)
Doran, 2011 (England)[7]	CPRD	**Incentive introduction** (multiple QOF indicators in 2004)<br><br>**Never incentivised** indicators (evaluated before and after QOF implementation in 2004)	ITSA (January 2000 to December 2007)	148 practices<br>Mean list size of 7117 (SD 3441)	No specific inclusion criteria for patients<br><br>A random selection of 4500 patients registered for at least one day between 1 January 2000 and 31 December 2007 was drawn from each practice<br><br>For practices with fewer than 4501 patients, all patients were selected<br><br>Patients were included in the sample for a year provided they were registered with their practice for the entire year	Based on annual data, to investigate whether the QOF for UK general practitioners led them to neglect activities not included in the scheme.	Quantitative synthesis<br>Used one and three year impact as reported by original paper
Fichera, 2016 (England)[8]	Health Survey for England	**Incentive introduction** (lifestyle related QOF indicators in 2004)	Sharp regression discontinuity analysis (1997 to 2009)	Number of practices not applicable<br>Sample size: 32102	Individuals reporting at least one condition incentivised by the QOF, including cancer, diabetes, other endocrine problems, mental health, stroke, heart attack/angina, hypertension/high blood pressure,	Based on data with different choices of bandwidths, to explore whether the introduction of QOF affected the population's weight, smoking and drinking behaviours.	Narrative synthesis (outcomes only indirectly related to incentivisation)

					bronchitis, asthma, and other respiratory problems		
Gallagher, 2015 (England)[9]	CPRD	**Incentive introduction** (diabetes indicators in QOF in 2004)	ITSA (1999 to 2008)	516 practices<br>Sample size: 100000, with incident (newly diagnosed) cases of 21197 and prevalent (previously diagnosed) cases of 78803	Patients with a diagnosis of Type 2 diabetes over a 10-year period, 1999–2008. Type 2 diabetes was defined within the dataset as the presence of a clinical diagnostic code for Type 2 diabetes or unspecified diabetes, and diagnosis at age ≥35 years	Based on annual data from 100000 people with 10000 randomly selected from each year, to determine whether the QOF financial incentives for tight glycaemic control increased the rate at which people with newly diagnosed Type 2 diabetes were started on anti-diabetic medication.	Narrative synthesis (outcomes only indirectly related to incentivisation)
Gilbert, 2019 (England)[10]	CPRD	**Incentive introduction** (diabetes indicators in QOF in 2004)	ITSA (2000 to 2015)	125 practices<br>Sample size: 37065	Patients with diabetes identified using a broader list of Read codes than that captured by QOF registers. Their data were collected if:<br>1) they were registered with the practice and diagnosed with diabetes prior to the end of that year;<br>2) did not transfer out or die until after the start of that year;<br>3) the practice had their last collection date after the end of that year; and<br>4) the practice data were at research standard	Based on annual data, to assess the impact of the QOF on general practitioner consultation rates for patients with diabetes.	Narrative synthesis (outcomes only indirectly related to incentivisation)
Hardoon, 2016 (UK)[11]	THIN	**Incentive introduction** (QOF serious mental illness review indicators SMI in 2004, specific screening indicators in 2006, addition of alcohol screening in 2011)	Retrospective cohort (2000 to 2013)	484 practices<br>Sample size: 14051 with bipolar disorder, and 90023 age-sex-practice matched without	Patients aged 18-99 years with or without bipolar disorder	To compare alcohol recording in people with (incentivised) and without (not-incentivised) bipolar disorder in various 2 year periods reflecting changing QOF incentivisation	Narrative synthesis (not enough data points for ITSA)
Harrison, 2014 (England)[12]	Hospital Episode Statistics	**Incentive introduction** (multiple QOF indicators in 2004)	Controlled longitudinal (1998 to 2011)	6975 practices<br>Mean list size of 6264	Patients admitted to hospitals as “emergency admissions” (the first episode in a spell of care, coded as an emergency, and admitted from a source other than another hospital ward or outpatient clinic)	Based on annual data, to estimate the impact of QOF on emergency hospital admissions for ambulatory care sensitive conditions.	Narrative synthesis (outcomes only indirectly related to incentivisation)
James, 2014 (UK)[13]	THIN	**Incentive introduction** (COPD indicators in QOF in 2004)	Retrospective cohort (2000 to 2009)	419 practices<br>Sample size: 92576	Patients with a diagnosis of chronic obstructive pulmonary disease based on relevant QOF Read codes	Based on annual data, to describe changes in the management and outcomes of patients with chronic obstructive pulmonary disease in	Narrative synthesis (outcomes only indirectly related to incentivisation)

						UK general practice between 2000 and 2009.	
Kendrick, 2015 (England)[14]	CPRD	**Incentive introduction** (QOF indicator for assessment of depression severity at diagnosis in 2006)	ITSA (2003 to 2013)	142 practices<br>Sample size: 293596	Patients who between 1 April 2003 and 31 March 2013 had clinical or referral events recorded which included a Read code for non-psychotic depressive symptoms or diagnoses, or for assessment using depression symptom questionnaires	Based on quarterly data, to determine how general practitioners' rates of recording of depression changed in England and explore possible effects of QOF introduction and recession.	Narrative synthesis (outcomes only indirectly related to incentivisation)
Khadjesari 2017 (UK)[15]	THIN	**Incentive introduction** (QOF serious mental illness review indicators SMI in 2004, specific screening indicators in 2006, addition of alcohol screening in 2011)	Retrospective cohort (2000 to 2013)	409 practices<br>Sample size: 34440 with schizophrenia, and 226984 age-sex-practice matched without	Patients aged 18-99 years with or without schizophrenia	To evaluate the impact of QOF incentives by comparing alcohol consumption in various two year periods matching changing QOF incentivisation, in people with (incentivised) and without (not-incentivised) schizophrenia	Narrative synthesis (not enough data points for ITSA)
Kontopantelis, 2015 (UK)[16]	CPRD	**Incentive introduction** (QOF serious mental illness indicators in 2004)	ITSA (2000 to 2012)	50 practices<br>Sample size: 346551 SMI cases matched to five individuals without SMI (1732755) on age, gender and general practice	Patients registered with a CPRD practice for the whole year and aged ≥18 with the Read code for SMI<br><br>Within each year, each SMI case was matched on age, sex, and practice to five randomly selected patients not associated with SMI up until that time point	Based on annual data, to examine the consultation rate patterns of patients with severe mental illness and whether they were impacted by the introduction of QOF.	Narrative synthesis (outcomes only indirectly related to incentivisation)
Kontopantelis, 2013 (UK)[17]	CPRD	**Incentive introduction** (QOF diabetes indicators in 2004)	ITSA (2000 to 2007)	148 practices<br>Sample size: 23920 diabetic patients	Patients aged ≥17 that had been registered with a selected practice for at least one day during the study period—were selected from practices with list sizes of 4500 or below<br><br>For larger practices, a random selection of 4500 patients was drawn from each practice. Patients with either type 1 or type 2 diabetes were identified using the relevant diagnostic codes	Based on annual data, to assess the effect of the incentives on recorded quality of care for diabetes patients and its variation by patient and practice characteristics.	Quantitative synthesis<br>New ITSA model fitted from data extracted from table in original paper
Kontopantelis, 2014 (UK)[18]	CPRD	**Incentive withdrawal** (8 QOF indicators in 2006 and 2011, 6 of which were process indicators where the	Multilevel mixed effects model of time series data (2004 to 2012)	452 practices<br>Sample size: 13772992	Patients registered with an included practice for the full year	Based on annual data, to investigate the effect of withdrawing incentives on recorded quality of care, in the context of QOF.	Narrative synthesis (processes of care examined remained incentivised by other indicators, so only partial

		linked intermediate outcome remained incentivised)					withdrawal)
Ma, 2020 (England, Scotland, and Wales)[19]	CPRD	**Incentive introduction** (QOF long-acting reversible contraception advice indicator in 2009)	ITSA (2004 to 2014)	>600 practices<br>Sample size: 3281667	Women aged 13 to 54 years registered with a primary care practice at any point that year that was deemed "up to standard" (when the practice met data quality and completeness criteria) in the previous year	Based on annual data, to examine the impact and equity of QOF on long-acting reversible contraception uptake and abortions.	Quantitative synthesis<br>Used one and *four* year impact as reported by original paper
Matias, 2024 (England)	CPRD	**Incentive withdrawal** (QOF serious mental illness record of BMI and cholesterol in 2014)<br><br>**Incentive reintroduction** (QOF serious mental illness record of BMI and alcohol consumption in 2019)	Difference-in-differences controlled for BP record (incentivised throughout)	Number of practices not reported (declines over time)<br><br>Sample size: 5635 patients with SMI in 2012, 3065 in 2018	Patients aged ≥18 years who were registered with a practice in CPRD GOLD at any time between April 2011 and March 2020, and had a diagnosis of schizophrenia, bipolar disorder, and other psychoses and other affective disorders documented in primary care	Based on annual data, to assess the impact of removing and reintroducing QOF financial incentives on uptake of three physical health checks (BMI, cholesterol, and alcohol consumption) for patients with serious mental illness	Quantitative synthesis for incentive withdrawal<br>New ITSA model fitted from data extracted from plot in original paper<br><br>Narrative synthesis for incentive reintroduction (not enough data points for ITSA)
McLintock, 2014 (Leeds, England)[20]	GP electronic health record data	**Incentive introduction** (QOF indicator for case finding for depression in patients with a diagnosis of coronary heart disease or diabetes 2006)	ITSA (2002 to 2012)	65 practices<br>Sample size: 37229 with diabetes and coronary heart disease targeted by case finding incentives, and 101008 patients with four other long-term conditions not targeted (hypertension, epilepsy, chronic obstructive pulmonary disease and asthma)	Patients aged ≥18 years	Based on monthly data, to evaluate the effects of QOF incentivised case finding for depression on diagnosis and treatment in targeted and non-targeted long-term conditions.	Narrative synthesis (non-linear trends in QOF incentivised outcome preclude new ITSA)
Millett, 2009 (UK)[21]	CPRD	**Incentive introduction** (QOF indicators for diabetes intermediate outcome control in 2004)	ITSA (1997 to 2005)	422 practices<br>Sample size: 154,945 people with diabetes (49970 in 1997, 105,065 in 2005)	Adults with type 1 or type 2 diabetes	Annual measurement of control of HbA1c (≤7.0%), blood pressure (≤140/80mmHg) and total cholesterol (≤5mmol/L)	Narrative synthesis (not enough data points for ITSA)
Minchin, 2018 (England)[22]	INLIQ	**Incentive withdrawal** (12 indicators in 2014)	ITSA (2010 to 2017)	2819 practices<br>>20 million registered patients	No specific inclusion criteria for patients	Based on annual data, to estimate the effects of removing incentives on changes in performance on quality-of-care measures.	Quantitative synthesis<br>Used one and three year impact as reported by original paper
Morales, 2023 (Scotland and	TQA in Scotland and	**Incentive withdrawal** (16 indicators in 2016)	ITSA (2013 to 2019)	979 practices in Scotland and 7921	Patients registered with included practices in Scotland or England for	Based on annual data, to determine whether the	Quantitative synthesis<br>Used one and three year

England)[23]	QOF public reporting in England			practices in England<br>Sample size: 5599171 in Scotland; 56270628 in England	the full year	withdrawal of QOF in primary care in Scotland in 2016 had an impact on selected recorded quality of care, compared with England where the scheme continued.	impact as reported by original paper
Pasvol, 2022 (UK)[24]	IMRD	**Incentive introduction** (QOF long-acting reversible contraception advice indicator in 2009)<br><br>**Incentive withdrawal** (QOF LARC indicator in 2014)	ITSA<br>(2000 to 2018)	797 practices<br>Sample size: 2705638	Women aged 15–49 years who contributed data to IMRD	Based on annual data, to describe temporal trends in non-barrier contraceptive prescribing in UK primary care for the period 2000–2018.	Quantitative synthesis<br>New ITSA model fitted from data extracted from plot in original paper
Ryan, 2016 (UK)[25]	World Health Organization Mortality Database	**Incentive introduction** (multiple QOF indicators in 2004)	Retrospective cohort<br>(1994 to 2010)	Number of practices not applicable<br>List or sample size not reported	Not applicable	Based on annual data, to test whether the QOF is associated with broad improvements in population health.	Narrative synthesis (outcomes only indirectly related to incentivisation)
Serumaga (2011)	THIN	**Incentive introduction** (QOF indicators for blood pressure measurement and control in hypertension in 2004)	ITSA (2000 to 2007)	358 practices<br>Sample size 470725	People permanently registered with a contributing practice, with a diagnosis of hypertension (defined somewhat more narrowly than in QOF)	Based on quarterly data, to determine immediate impact and change in trend for blood pressure record, blood pressure control, and number of anti-hypertensive medications.	Narrative synthesis (incompletely reported time-series data)
Sheppard, 2018 (England)[26]	CPRD	**Incentive introduction** (QOF hypertension indicators for lifestyle advice and stricter treatment targets 2009)<br><br>**Incentive withdrawal** (QOF lifestyle advice indicators 2013)	Retrospective cohort<br>(1998 to 2015)	Number of practices not reported<br>Sample size: 108843	Patients aged 18–74 years, registered at general practices contributing to the CPRD in England, and with stage 1 hypertension (blood pressure between 140/90 and 159/99mm Hg), no cardiovascular disease risk factors and no treatment	Based on monthly data, to determine the incidence of lifestyle advice and drug therapy in this population and whether secular trends were associated with policy changes.	Narrative synthesis (outcomes are measured as incidence of lifestyle advice per month, so cannot be quantitatively synthesised in terms of annual impact)
Simpson, 2011 (Scotland)[27]	Primary Care Clinical Informatics Unit Database	**Incentive introduction** (QOF hypertension indicators 2004)	Descriptive analysis of time-series data<br>(2001 to 2006)	315 practices<br>Sample size: 826973	Patients aged ≥40 years with a computer record of hypertension (Read code G2, G20 and below, G24–G2z) and no subsequent recording of a Read code indicating that their hypertension had resolved (Read code 21261 and 212K) at six time points (1 April 2001, 1 April 2002, 1 April 2003, 1 April 2004, 1 April 2005, and 1 April 2006).	Based on annual data, to investigate the impact of pay for performance on the management of patients with hypertension in Scottish primary care.	Quantitative synthesis<br>New ITSA model fitted from data extracted from plot in original paper

Sutton, 2010 (Scotland)[28]	Primary Care Clinical Informatics Unit Database	**Incentive introduction** (QOF multiple risk factor recording indicators in chronic conditions 2004)	Retrospective cohort (2000 to 2006)	315 practices<br>Sample size: 391323	Patients aged ≥45 years with coronary heart disease, chronic obstructive pulmonary disease, diabetes, hypertension, or stroke	To estimate the intended and unintended consequences of QOF in blood pressure, smoking status, cholesterol, body mass index, and alcohol consumption using dynamic panel probit models estimated on annual individual patient records.	Narrative synthesis (not enough data points for ITSA)
Szatkowski, 2016 (England)[29]	THIN	**Incentive introduction** (QOF smoking indicator revision 2012)	Autoregressive integrated moving average (ARIMA) model (2004 to 2013)	315 practices<br>Sample size: 3337881 on average each month	Patients aged >16 years who contributed data to THIN each month from April 2004 (when the QOF was implemented) to March 2013 (1 year after the revision to the smoking targets)	Based on monthly data, to quantify the impact of this new recommendation and payment on indicators of smoking cessation activity.	Narrative synthesis (could not accurately extract data from plot)
Taggar, 2012 (UK)[30]	THIN	**Incentive introduction** (QOF smoking indicator implementation 2004 and revision 2006 and 2008)	Regression analysis of individual characteristics associated with outcome in each year (2000 to 2008)	446 practices<br>Sample size: 1998631 in 2002; 2053840 in 2004; 2149026 in 2008	Patients aged >15 years who were registered in THIN on an index date of 1 April in each year	Based on annual data, to investigate the association between smoking-related QOF targets and such recording, and the factors which influence these clinical activities.	Quantitative synthesis<br>New ITSA model fitted from data extracted from plot in original paper
Wilson, 2017 (UK)[31]	CPRD	**Incentive introduction** (QOF serious mental illness review indicators SMI in 2004, specific screening indicators in 2006, addition of alcohol in 2011)	ITSA (1996 to 2014)	232 practices in 1995/6; 595 practices in 2004/5; 530 practices in 2013/14<br>Sample size: 67239 SMI patients; 359951 patients without SMI	Case group: patients aged ≥35 years with a life-time diagnosis of SMI<br><br>Control group: unmatched, randomly selected patients aged ≥35 years without SMI who had a period of continuous registration during the study period, aiming for a minimum ratio of controls to cases of 5:1	Based on annual data, to assess the QOF's impact on detection and treatment of cardiovascular risk factors in people with SMI.	Narrative synthesis (outcomes not suitable for quantitative synthesis)

PACT: Prescribing Analysis and Cost data; CPRD: Clinical Practice Research Datalink dataset; IMRD: IQVIA Medical Research Data; INLIQ: NHS England Indicators No Longer in QOF dataset; TQA: NHS Scotland Transitional Quality Arrangements dataset; ITSA: Interrupted time series analysis; SD: Standard deviation; SMI: Severe mental illness; THIN: The Health Improvement Network; QOF: Quality and Outcomes Framework; UK: United Kingdom

Supplementary Table B: Studies excluded after full-text review

Study	Reason for exclusion
Adam R. 'Personal care' and general practice medicine in the UK: A qualitative interview study with patients and general practitioners. *Osteopath Med Prim Care* 2007; 1.	Wrong intervention (not QOF)
Adams E, Boulton M, Rose P, et al. Views of cancer care reviews in primary care: a qualitative study. *BJGP* 2011; 61(585): 173-82.	Wrong study design
Alabbadi I, Crealey G, Turner K, et al. Statin prescribing in Northern Ireland and England pre and post introduction of the quality and outcomes framework. *Pharm World Sci* 2010; 32(1): 43-51.	Wrong outcomes
Alshamsan R, Lee JT, Majeed A, Netuveli G, Millett C. Effect of a UK pay-for-performance program on ethnic disparities in diabetes outcomes: interrupted time series analysis. *Ann Fam Med* 2012; 10(3): 228-34.	Wrong study design (study population is not consistent over time)[a]
Bottle A, Gnani S, Saxena S, Aylin P, Mainous AG, 3rd, Majeed A. Association between quality of primary care and hospitalization for coronary heart disease in England: national cross-sectional study. *J Gen Intern Med* 2008; 23(2): 135-41.	Wrong study design (no data pre-incentivisation)
Brettell R, Soljak M, Cecil E, Cowie MR, Tuppin P, Majeed A. Reducing heart failure admission rates in England 2004-2011 are not related to changes in primary care quality: national observational study. *Eur J Heart Fail* 2013; 15(12): 1335-42.	Wrong study design (no data pre-incentivisation)
Campbell S, Reeves D, Kontopantelis E, Middleton E, Sibbald B, Roland M. Quality of primary care in England with the introduction of pay for performance. *N Engl J Med* 2007; 357(2): 181-90.	Duplicate report[b]
Chenot R. Linking physicians' pay to the quality of care: The British experiment. *ZFA (Stuttgart)* 2007; 83(6): 242-6.	Wrong study design (no data pre-incentivisation)
Close J, Fosh B, Wheat H, et al. Longitudinal evaluation of a countywide alternative to the Quality and Outcomes Framework in UK General Practice aimed at improving Person Centred Coordinated Care. *BMJ Open* 2019; 9(7): e029721.	Wrong intervention (not QOF)
Coleman T, Lewis S, Hubbard R, Smith C. Impact of contractual financial incentives on the ascertainment and management of smoking in primary care. *Addiction* 2007; 102(5): 803-8.	Wrong study design (no formal analysis, only one year of post-intervention data))
Coleman T. Do financial incentives for delivering health promotion counselling work? Analysis of smoking cessation activities stimulated by the quality and outcomes framework. *BMC Public Health* 2010; 10(100968562): 167.	Not a research paper (debate’ article, no new data)
Crawley D, Ng A, Mainous AG, III, et al. Impact of pay for performance on quality of chronic disease management by social class group in England. *J R Soc Med* 2009; 102(3): 103-7.	Wrong study design (doesn’t report population impact)
Crinson I, Shaw A, Durrant R, De Lusignan S, Williams B. Coronary heart disease and the management of risk: Patient perspectives of outcomes associated with the clinical implementation of the National Service Framework targets. *Health Risk Soc* 2007; 9(4): 359-73.	Wrong study design (qualitative study)
Dhoul N, de Lusignan S, Dmitrieva O, Stevens P, O'Donoghue D. Quality achievement and disease prevalence in primary care predicts regional variation in renal replacement therapy (RRT) incidence: an ecological study. *Nephrol Dial Transplant* 2012; 27(2): 739-46.	Wrong study design (no data pre-incentivisation)
Downing A, Rudge G, Cheng Y, Tu Y-K, Keen J, Gilthorpe MS. Do the UK government's new Quality and Outcomes Framework (QOF) scores adequately measure primary care performance? A cross-sectional survey of routine healthcare data. *BMC Health Serv Res* 2007; 7(101088677): 166.	Wrong study design (no data pre-incentivisation)
Feakins B, Oke J, McFadden E, et al. Trends in kidney function testing in UK primary care since the introduction of the quality and outcomes framework: a retrospective cohort study using CPRD. *BMJ Open* 2019; 9(6): e028062.	Wrong study design (no data pre-incentivisation)
Fearn S, Larner AJ. Have Quality and Outcomes Framework Depression Indicators changed referrals from primary care to a dedicated memory clinic? *Ment Health Fam Med* 2010; 6(3): 129-32.	Wrong study design (single clinic audit)
Fleetcroft R, Parekh-Bhurke S, Howe A, Cookson R, Swift L, Steel N. The UK pay-for-performance programme in primary care: estimation of population mortality reduction. *BJGP* 2010; 60(578): e345-52.	Wrong study design (modelling mortality impact)

Gabel F, Chambers R, Cox T, Listl S, Maskrey N. An evaluation of a multifaceted, local Quality Improvement Framework for long-term conditions in UK primary care. *Fam Pract* 2019; 36(5): 607-13.	Wrong intervention (not QOF)
Gadsby R, Young B. Do changes to Quality and Outcomes Framework diabetes clinical indicators affect care as measured in the National Diabetes Audit? *Diabet Med* 2016; 33(12): 1748-9.	Wrong study design (no data pre-incentivisation)
Grigoroglou C, Munford L, Webb R, et al. Impact of a national primary care pay-for-performance scheme on ambulatory care sensitive hospital admissions: a small-area analysis in England. *BMJ Open* 2020; 10(9): e036046.	Wrong study design (no data pre-incentivisation)
Grigoroglou C, Munford L, Webb RT, et al. Association between a national primary care pay-for-performance scheme and suicide rates in England: spatial cohort study. *BJPsych* 2018; 213(4): 600-8.	Wrong study design (no data pre-incentivisation)
Gulliford MC, Ashworth M, Robotham D, Mohiddin A. Achievement of metabolic targets for diabetes by English primary care practices under a new system of incentives. *Diabet Med* 2007; 24(5): 505-11.	Wrong study design (pre- and post-incentivisation data is inconsistent)
Gunn LH, McKay AJ, Molokhia M, et al. Associations between attainment of incentivised primary care indicators and emergency hospital admissions among type 2 diabetes patients: a population-based historical cohort study. *J R Soc Med* 2021; 114(6): 299-312.	Wrong study design (no data pre-incentivisation)
Gunn LH, Vamos EP, Majeed A, et al. Associations between attainment of incentivized primary care indicators and incident lower limb amputation among those with type 2 diabetes: a population-based historical cohort study. *BMJ Open Diabetes Res Care* 2021; 9(1).	Wrong study design (no data pre-incentivisation)
Hamilton FL, Bottle A, Vamos EP, et al. Impact of a pay-for-performance incentive scheme on age, sex, and socioeconomic disparities in diabetes management in UK primary care. *J Ambul Care Manage* 2010; 33(4): 336-49.	Wrong study design (doesn't report population impact)
Hamilton FL, Laverty AA, Gluvajic D, et al. Effect of financial incentives on delivery of alcohol screening and brief intervention (ASBI) in primary care: longitudinal study. *J Public Health (Oxf)* 2014; 36(3): 450-9.	Wrong intervention (not QOF)
Hamilton FL, Laverty AA, Vamos EP, Majeed A, Millett C. Effect of financial incentives on ethnic disparities in smoking cessation interventions in primary care: cross-sectional study. *J Public Health (Oxf)* 2013; 35(1): 75-84.	Wrong study design (no data pre-incentivisation)
Hardy B, Szatkowski L, Tata LJ, Coleman T, Dhalwani NN. Smoking cessation advice recorded during pregnancy in United Kingdom primary care. *BMC Fam Pract* 2014; 15(100967792): 21.	Wrong patient population
Jaiveer PK, Jaiveer S, Jujjavarapu SB, et al. Improvements in clinical diabetes care in the first year of the new General Medical Services contract in the UK. *Br J Diabetes Vasc Dis* 2006; 6(1): 45-8.	Wrong study design (uncontrolled single timepoint before and after)
Karunaratne K, Stevens P, Irving J, et al. The impact of pay for performance on the control of blood pressure in people with chronic kidney disease stage 3-5. *Nephrol Dial Transplant* 2013; 28(8): 2107-16.	Wrong study design (uncontrolled single timepoint before and after)
Kasteridis P, Mason A, Goddard M, et al. Risk of Care Home Placement following Acute Hospital Admission: Effects of a Pay-for-Performance Scheme for Dementia. *PLoS One* 2016; 11(5): e0155850.	Wrong study design (no data pre-incentivisation)
Khan N, Rudoler D, McDiarmid M, Peckham S. A pay for performance scheme in primary care: Meta-synthesis of qualitative studies on the provider experiences of the quality and outcomes framework in the UK. *BMC Fam Pract* 2020; 21(1): 142.	Wrong study design
Khedmati Morasae E, Rose TC, Gabbay M, et al. Evaluating the Effectiveness of a Local Primary Care Incentive Scheme: A Difference-in-Differences Study. *Med Care Res Rev* 2022; 79(3): 394-403.	Wrong intervention
Lee JT, Netuveli G, Majeed A, Millett C. The effects of pay for performance on disparities in stroke, hypertension, and coronary heart disease management: interrupted time series study. *PLoS One* 2011; 6(12): e27236.	Wrong study design (study population is not consistent over time)[a]
Liu D, Green E, Kasteridis P, et al. Incentive schemes to increase dementia diagnoses in primary care in England: a retrospective cohort study of unintended consequences. *BJGP* 2019; 69(680): e154-e63.	Wrong intervention (not QOF)
MacBride-Stewart SP, Elton R, Walley T. Do quality incentives change prescribing patterns	Wrong outcomes

in primary care? An observational study in Scotland. *Fam Pract* 2008; 25(1): 27-32.	
Mackintosh M, Aldridge RW, Rossor M, et al. Dementia recognition, diagnosis, and treatment in the UK, 1997-2017: a change-point analysis. *Lancet* 2019; 394(Supplement 2): S70.	Not a research paper (conference abstract)
Maisey S, Steel N, Marsh R, Gillam S, Fleetcroft R, Howe A. Effects of payment for performance in primary care: qualitative interview study. *J Health Serv Res Policy* 2008; 13(3): 133-9.	Wrong study design (qualitative study)
McKay AJ, Gunn LH, Vamos EP, et al. Associations between attainment of incentivised primary care diabetes indicators and mortality in an English cohort. *Diabetes Res Clin Pract* 2021; 174(ebi, 8508335): 108746.	Wrong study design (no pre-incentivisation data)
McManus E, Elliott J, Meacock R, Wilson P, Gellatly J, Sutton M. The effects of structure, process and outcome incentives on primary care referrals to a national prevention programme. *Health Econ* 2021; 30(6): 1393-416.	Wrong intervention (not QOF)
Mendonca SC, Saunders CL, Lund J, Mant J, Edwards D. Temporal trends in incidence of atrial fibrillation in primary care records: a population-based cohort study. *BMJ Open* 2020; 10(12): e042518.	Wrong study design (no pre-incentivisation data)
Millett C, Gray J, Saxena S, Netuveli G, Khunti K, Majeed A. Ethnic disparities in diabetes management and pay-for-performance in the UK: the Wandsworth Prospective Diabetes Study. *PLoS Med* 2007; 4(6): e191.	Wrong study design (uncontrolled single timepoint before and after)
Millett C, Gray J, Saxena S, Netuveli G, Majeed A. Impact of a pay-for-performance incentive on support for smoking cessation and on smoking prevalence among people with diabetes. *CMAJ* 2007; 176(12): 1705-10.	Wrong study design (no pre-incentivisation data)
Millett C, Gray J, Wall M, Majeed A. Ethnic disparities in coronary heart disease management and pay for performance in the UK. *J Gen Intern Med* 2009; 24(1): 8-13.	Wrong study design (study population is not consistent over time)[a]
Millett C, Netuveli G, Saxena S, Majeed A. Impact of pay for performance on ethnic disparities in intermediate outcomes for diabetes: a longitudinal study. *Diabetes Care* 2009; 32(3): 404-9.	Wrong study design (uncontrolled single timepoint before and after)
Mindell J, Aresu M, Zaninotto P, Falaschetti E, Poulter N. Improving lipid profiles and increasing use of lipid-lowering therapy in England: results from a national cross-sectional survey - 2006. *Clin Endocrinol (Oxf)* 2011; 75(5): 621-7.	Wrong study design (study population is not consistent over time)[c]
Minshall I, Buckels L, Cox P, et al. UK general practice: Continuing to fail in the care of people with epilepsy? *Epilepsy Behav* 2021; 124(100892858): 108354.	Wrong study design (study population is not consistent over time)[a]
Murray J, Saxena S, Millett C, Curcin V, de Lusignan S, Majeed A. Reductions in risk factors for secondary prevention of coronary heart disease by ethnic group in south-west London: 10-year longitudinal study (1998-2007). *Fam Pract* 2010; 27(4): 430-8.	Wrong study design (study population is not consistent over time)[a]
Norbury M, Fawkes N, Guthrie B. Impact of the GP contract on inequalities associated with influenza immunisation: a retrospective population-database analysis. *BJGP* 2011; 61(588): e379-85.	Wrong study design (uncontrolled single timepoint before and after)
O'Donnell A, Angus C, Hanratty B, Hamilton FL, Petersen I, Kaner E. Impact of the introduction and withdrawal of financial incentives on the delivery of alcohol screening and brief advice in English primary health care: an interrupted time-series analysis. *Addiction* 2020; 115(1): 49-60.	Wrong intervention (not QOF)
O'Donnell A, Haighton C, Chappel D, Shevills C, Kaner E. Impact of financial incentives on alcohol intervention delivery in primary care: a mixed-methods study. *BMC Fam Pract* 2016; 17(1): 165.	Wrong intervention (not QOF)
Pandya A, Doran T, Zhu J, Walker S, Arntson E, Ryan AM. Modelling the cost-effectiveness of pay-for-performance in primary care in the UK. *BMC Med* 2018; 16(1): 135.	Wrong study design (cost-effectiveness modelling)
Pape UJ, Huckvale K, Car J, Majeed A, Millett C. Impact of 'stretch' targets for cardiovascular disease management within a local pay-for-performance programme. *PLoS One* 2015; 10(3): e0119185.	Wrong intervention (not QOF)
Peckham S, Hann A. General practice and public health: Assessing the impact of the new GMS contract. *Crit Public Health* 2008; 18(3): 347-56.	Not a research paper (narrative review, no new data)
Phillips LA, Phillips BM, Meran S, et al. The long-term impact of eGFR reporting on referral	Wrong outcomes

patterns. *Eur J Intern Med* 2014; 25(1): 97-101.	
Quinn L, Hosier I, Adderley NJ, Marshall T. An interrupted time-series analysis of the effects of withdrawal of financial incentives on diagnosis of atrial fibrillation as resolved. Does withdrawal of an incentive reverse its unintended effects? *BJGP Open* 2022; 6(4).	Wrong outcomes
Saxena S, Car J, Eldred D, Soljak M, Majeed A. Practice size, caseload, deprivation and quality of care of patients with coronary heart disease, hypertension and stroke in primary care: national cross-sectional study. *BMC Health Serv Res* 2007; 7(101088677): 96.	Wrong study design (no pre-incentivisation data)
Shaikh H, Hui E, Qureshi A. Effect of the nGMS contract on diabetes control. *Diabetes Prim Care* 2007; 9(2): 122-4.	Wrong patient population (type 2 diabetes attending hospital clinic)
Simpson CR, Hannaford PC, Lefevre K, Williams D. Effect of the UK incentive-based contract on the management of patients with stroke in primary care. *Stroke* 2006; 37(9): 2354-60.	Wrong study design (uncontrolled single timepoint before and after)
Smith CJP, Gribbin J, Challen KB, Hubbard RB. The impact of the 2004 NICE guideline and 2003 General Medical Services contract on COPD in primary care in the UK. *QJM* 2008; 101(2): 145-53.	Wrong study design (uncontrolled single timepoint before and after)
Smith T, Fell C, Otete H, Chauhan U. GP incentives to design hypertension and atrial fibrillation local quality-improvement schemes: a controlled before-after study in UK primary care. *BJGP* 2019; 69(687): e689-e96.	Wrong intervention (not QOF)
Steel N, Maisey S, Clark A, Fleetcroft R, Howe A. Quality of clinical primary care and targeted incentive payments: an observational study. *BJGP* 2007; 57(539): 449-54.	Wrong study design (uncontrolled single timepoint before and after)
Sutton M, McLean G. Determinants of primary medical care quality measured under the new UK contract: cross sectional study. *BMJ* 2006; 332(7538): 389-90.	Wrong study design (no pre-incentivisation data)
Szatkowski L, McNeill A, Lewis S, Coleman T. A comparison of patient recall of smoking cessation advice with advice recorded in electronic medical records. *BMC Public Health* 2011; 11(100968562): 291.	Wrong outcomes
Tahrani AA, McCarthy M, Godson J, et al. Diabetes care and the new GMS contract: the evidence for a whole county. *BJGP* 2007; 57(539): 483-5.	Wrong study design (indicators are measured in different ways before and after QOF implementation)
Tahrani AA, McCarthy M, Godson J, et al. Impact of practice size on delivery of diabetes care before and after the Quality and Outcomes Framework implementation. *BJGP* 2008; 58(553): 576-9.	Wrong study design (doesn't report population impact)
Vamos EP, Pape UJ, Bottle A, et al. Association of practice size and pay-for-performance incentives with the quality of diabetes management in primary care. *CMAJ* 2011; 183(12): E809-16.	Wrong study design (doesn't report population impact)
Whalley D, Gravelle H, Sibbald B. Effect of the new contract on GPs' working lives and perceptions of quality of care: a longitudinal survey. *BJGP* 2008; 58(546): 8-14.	Wrong study design (no pre-incentivisation data)
Williams PH, de Lusignan S. Does a higher 'quality points' score mean better care in stroke? An audit of general practice medical records. *Inform Prim Care* 2006; 14(1): 29-40.	Wrong study design (no pre-incentivisation data)

a. Study population is defined as a cross-section post-QOF implementation, and time-series is for these patients, so not repeated cross-sectional/consistent ascertainment of whole population at each timepoint
b. Included study Campbell 2009 reports same data with an additional time point
c. One timepoint before and two after QOF implementation, but second after timepoint is for a subset of older patients

Supplementary Table C. Risk of bias assessment of included studies

Author, year	Type of synthesis used in	Methodological quality criteria for quantitative non-randomised studies				
		Representative participants	Appropriate measurements	Complete outcome data	Accounted confounders	Intended intervention
Arrowsmith (2014)	Quantitative	Yes	Yes	Yes	Yes	Yes
Calvert (2009)	Quantitative	Yes	Yes	Yes	Yes	Yes
Campbell (2009)	Narrative	Yes	Yes	Yes	No[b]	Yes
Doran (2011)	Quantitative	Yes	Yes	Yes	Yes	Yes
Fichera (2016)	Narrative	Yes	Yes	Yes	Yes	Yes
Gallagher (2015)	Narrative	Yes	Yes	Yes	Yes	Yes
Gilbert (2019)	Narrative	Yes	Yes	Yes	Yes	Yes
Hardoon (2016)	Narrative	Yes	Yes	Yes	No[c]	Yes
Harrison (2014)	Narrative	Yes	Yes	Yes	Yes	Yes
James (2014)	Narrative	Yes	Yes	Yes	Yes	Yes
Kendrick (2015)	Narrative	Yes	Yes	Yes	Yes	Yes
Khadjesar (2017)	Narrative	Yes	Yes	Yes	No[c]	Yes
Kontopantelis (2015)	Narrative	Yes	Yes	Yes	Yes	Yes
Kontopantelis (2013)	Quantitative	Yes	Yes	Yes	Yes	Yes
Kontopantelis (2014)	Narrative	Yes	Yes	Yes	No[d]	Partially[e]
Ma (2020)	Narrative	Yes	Yes	Yes	Yes	Yes
Matias (2024)	Both	Yes	Yes	Yes	Yes[f]	Yes
McLintock (2014)	Narrative	No[a]	Yes	Yes	Yes	Yes
Millett (2009	Narrative	Yes	Yes	Yes	No[g]	Yes
Minchin (2018)	Quantitative	Yes	Yes	Yes	Yes	Yes
Morales (2023)	Quantitative	Yes	Yes	Yes	Yes	Yes
Pasvol (2022)	Quantitative	Yes	Yes	Yes	Yes	Yes
Ryan (2016)	Narrative	Yes	Yes	Yes	Yes	Yes
Serumaga (2011)	Narrative	Yes	Yes	Yes	Yes	Yes
Sheppard (2018)	Narrative	Yes	Yes	Yes	Yes	Yes
Simpson (2011)	Quantitative	Yes	Yes	Yes	Yes	Yes
Sutton (2010)	Narrative	Yes	Yes	Yes	No[g]	Yes
Szatkowski (2016)	Narrative	Yes	Yes	Yes	Yes	Yes
Taggar (2012)	Quantitative	Yes	Yes	Yes	Yes	Yes
Wilson (2017)	Narrative	Yes	Yes	Yes	Yes	Yes

a. Analysis is in a subset of practices in one city/region

b. Two time points before and two time points after incentives introduced

c. Outcome is record of alcohol consumption in people with serious mental illness but incentivisation is complex; incentives for 'annual review' 2004/5 to 2005/6, for 'annual review including lifestyle review' 2006/7 to 2010/2011, and specifically for alcohol record 2011/12 onwards. Only two datapoints after specific incentivisation.

d. Two indicators withdrawn in April 2006 have only two pre-withdrawal time points; Six indicators withdrawn in April 2011 have only one post-withdrawal time point.

e. One indicator withdrawn in April 2006 and six indicators withdrawn in April 2011 were for processes of care (eg record of blood pressure) where the process remained incentivised by a linked outcome indicator (eg blood pressure control indicator assumes that 'not recorded' is failed BP target). One indicator (flu immunisation in people with asthma) was not affected by this.

f. For incentive withdrawal, three time points before and five after; for incentive re-introduction only one time point after but analysis is controlled (difference-in-differences with a continuously incentivised process indicator in the same population)
g. Only two time points after incentive introduction

Supplementary Table D: Interrupted time-series analysis models for quality of care indicators where new modelling done

Study and indicator	Incentive introduction/ withdrawal, or never incentivised	Intercept<br><br>Percentage point (95% CI)	Pre-intervention trend<br>Percentage point change per unit of time (95% CI)	Step-change at time of intervention<br>Percentage point change (95% CI)	Change in trend after time of intervention<br>Percentage point change per unit of time (95% CI)
Arrowsmith, 2014 - new ITSA model[b]					
LARC prescribing rate[d]	Introduction	2.0 (1.9 to 2.0)	-0.1 (-0.02 to 0.004)	0.1 (0.04 to 0.2)	0.04 (0.03 to 0.05)
Calvert, 2009 - new ITSA model[a]					
Type 1 diabetes BMI record	Introduction	27.9 (12.8 to 43.1)	11.4 (1.4 to 21.3)	15.4 (-7.9 to 38.6)	-9.5 (12.8 to 43.1)
Type 1 diabetes smoking status record	Introduction	62.7 (54.1 to 71.3)	7.2 (1.6 to 12.8)	6.6 (-6.7 to 19.8)	-6.6 (54.1 to 71.3)
Type 1 diabetes HbA1c record	Introduction	57.1 (49.7 to 64.5)	8.3 (3.5 to 13.2)	3.0 (-8.3 to 14.3)	-8.1 (49.7 to 64.5)
Type 1 diabetes BP record	Introduction	68.4 (67.1 to 69.8)	5.9 (5.0 to 6.8)	4.0 (2.0 to 6.1)	-5.7 (-6.5 to -4.8)
Type 1 diabetes eGFR record	Introduction	44.7 (36.7 to 52.7)	8.9 (3.6 to 14.2)	8.4 (-4.2 to 20.9)	-7.2 (-12.8 to -1.6)
Type 1 diabetes cholesterol record	Introduction	48.7 (41.6 to 55.8)	8.0 (3.3 to 12.6)	7.4 (-3.6 to 18.4)	-7.2 (-12.1 to -2.4)
Type 2 diabetes BMI record	Introduction	43.5 (31.0 to 55.9)	11.3 (3.2 to 19.5)	7.2 (-11.7 to 26.1)	-10.2 (-18.4 to -2.0)
Type 1 diabetes smoking cessation advice record	Introduction	20.5 (-12.2 to 53.3)	13.4 (-8.0 to 34.8)	21.8 (-29.2 to 71.8)	-11.9 (-33.8 to 10.1)
Type 1 diabetes retinopathy screening record	Introduction	25.3 (6.5 to 44.0)	12.5 (0.2 to 24.8)	9.8 (-18.9 to 38.4)	-9.3 (-21.8 to 3.2)
Type 1 diabetes peripheral pulses record	Introduction	6.1 (-12.7 to 24.8)	9.9 (-2.4 to 22.2)	33.0 (3.3 to 62.8)	-7.7 (-21.3 to 5.9)
Type 1 diabetes neuropathy test record	Introduction	2.8 (-16.0 to 21.5)	9.7 (-2.6 to 22.0)	35.8 (5.9 to 65.8)	-7.0 (-16.0 to 21.5)
Type 1 diabetes microalbuminuria record	Introduction	9.9 (-0.1 to 20.0)	7.7 (1.1 to 14.2)	24.5 (8.6 to 40.3)	-3.8 (-11.0 to 3.5)
Type 1 diabetes HbA1c≤7.5%	Introduction	15.7 (12.2 to 19.2)	2.2 (-0.1 to 4.5)	2.3 (-3.8 to 8.3)	-1.5 (-4.5 to 1.5)
Type 1 diabetes HbA1c≤10%	Introduction	48.2 (41.5 to 54.9)	7.3 (2.9 to 11.7)	3.0 (-7.2 to 13.2)	-7.0 (41.5 to 54.9)
Type 1 diabetes BP≤145/85mmHg	Introduction	44.2 (43.4 to 45.0)	5.8 (5.3 to 6.3)	6.1 (3.2 to 9.1)	-3.4 (-5.2 to -1.6)
Type 1 diabetes cholesterol ≤5mmol/l	Introduction	25.4 (20.4 to 30.3)	7.5 (4.2 to 10.7)	8.2 (0.2 to 16.2)	-4.0 (20.4 to 30.3)
Type 1 diabetes ACEI/ARB for microalbuminuria	Introduction	73.8 (71.7 to 76.0)	-2.5 (-3.9 to -1.1)	9.5 (6.1 to 12.8)	5.2 (3.7 to 6.7)
Type 1 diabetes flu immunisation record	Introduction	49.8 (40.0 to 59.6)	1.5 (-4.9 to 8.0)	15.6 (0.02 to 31.1)	0.3 (-6.8 to 7.4)
Type 2 diabetes smoking status record	Introduction	71.8 (65.7 to 78.0)	6.3 (2.3 to 10.3)	3.8 (-5.7 to 13.2)	-6.2 (-10.4 to -2.0)
Type 2 diabetes HbA1c record	Introduction	71.9 (68.2 to 75.5)	5.6 (3.3 to 8.0)	-0.3 (-5.8 to 5.3)	-6.2 (-8.6 to 3.8)
Type 2 diabetes BP record	Introduction	85.5 (84.7 to 86.3)	2.7 (2.2 to 3.2)	0.9 (-0.3 to 2.2)	-2.7 (-3.3 to -2.2)
Type 2 diabetes eGFR record	Introduction	62.8 (56.9 to 68.7)	8.1 (4.2 to 12.0)	3.5 (-5.5 to 12.6)	-7.6 (-11.6 to -3.7)
Type 2 diabetes cholesterol record	Introduction	67.4 (64.2 to 70.6)	6.6 (4.5 to 8.7)	1.8 (-3.2 to 6.8)	-6.2 (-8.4 to -4.0)
Type 2 diabetes smoking cessation advice record	Introduction	24.5 (0.5 to 48.5)	16.0 (0.2 to 31.7)	14.7 (-21.9 to 51.4)	15.3 (-31.4 to 0.7)
Type 2 diabetes retinopathy screening record	Introduction	39.4 (31.0 to 47.9)	9.5 (3.9 to 15.0)	6.0 (-7.4 to 19.4)	-7.3 (-13.4 to -1.1)

Type 2 diabetes peripheral pulses record	Introduction	15.9 (-3.4 to 35.2)	11.4 (-1.2 to 24.0)	26.5 (-3.4 to 56.4)	-9.4 (-22.7 to 3.9)
Type 2 diabetes neuropathy test record	Introduction	9.6 (-11.3 to 30.5)	12.1 (-1.6 to 25.8)	30.3 (-2.2 to 62.8)	-10.1 (-11.3 to 30.5)
Type 2 diabetes microalbuminuria record	Introduction	14.2 (8.6 to 19.8)	10.7 (7.0 to 14.4)	22.1 (10.9 to 33.3)	-6.8 (-12.8 to -0.8)
Type 2 diabetes HbA1c≤7.5%	Introduction	39.4 (38.4 to 40.3)	5.0 (4.4 to 5.7)	1.3 (-0.8 to 3.3)	-3.4 (-4.5 to -2.3)
Type 2 diabetes HbA1c≤10%	Introduction	66.0 (62.9 to 69.1)	6.0 (3.9 to 8.0)	-0.3 (-5.1 to 4.4)	-6.3 (-8.4 to -4.2)
Type 2 diabetes BP≤145/85mmHg	Introduction	44.2 (43.5 to 44.9)	5.1 (4.7 to 5.6)	5.2 (4.0 to 6.3)	-2.3 (-2.8 to -1.7)
Type 2 diabetes cholesterol ≤5mmol/l	Introduction	33.1 (29.2 to 37.0)	9.6 (7.0 to 12.1)	5.1 (-10 to 11.3)	-5.8 (-1.0 to 11.3)
Type 2 diabetes ACEI/ARB for microalbuminuria	Introduction	62.4 (59.1 to 65.8)	7.0 (4.8 to 9.1)	-1.3 (-7.1 to 4.5)	-4.9 (-7.8 to 2.0)
Type 2 diabetes flu immunisation record	Introduction	61.6 (51.3 to 71.9)	3.0 (-3.7 to 9.8)	11.3 (-4.3 to 26.9)	-2.3 (-9.1 to 4.5)
Kontopantelis, 2013 - new ITSA model[a]					
All diabetes BMI record	Introduction	66.9 (65.5 to 68.3)	3.9 (3.4 to 4.4)	8.5 (7.4 to 9.7)	-3.1 (-37 to -2.4)
All diabetes smoking status record	Introduction	35.5 (35.2 to 35.9)	8.7 (8.6 to 8.8)	20.6 (18.6 to 22.6)	-8.6 (-10.0 to -7.3)
All diabetes HbA1c record	Introduction	71.4 (69.8 to 73.0)	5.9 (5.3 to 6.4)	-1.7 (-3.1 to -0.4)	-5.6 (-6.4 to -4.8)
All diabetes blood pressure record	Introduction	82.6 (81.9 to 83.3)	3.4 (3.2 to 3.6)	0,1 (-0.4 to 0.7)	-3.2 (-3.5 to -2.9)
All diabetes creatinine record	Introduction	52.0 (50.9 to 53.1)	10.4 (9.4 to 11.4)	-0.3 (-4.4 to 3.8)	-9.7 (-11.0 to -8.3)
All diabetes cholesterol record	Introduction	57.4 (57.3 to 57.6)	9.2 (9.2 to 9.3)	-1.5 (-2.7 to -0.4)	-8.7 (-9.4 to -7.9)
All diabetes smoking cessation advice record	Introduction	5.4 (4.6 to 6.1)	4.0 (3.7 to 4.2)	61.7 (58.2 to 65.3)	-1.0 (-3.4 to 1.3)
All diabetes retinopathy screening record	Introduction	41.6 (38.6 to 44.6)	6.8 (5.7 to 7.8)	4.0 (0.6 to 7.5)	-9.3 (-11.5 to -7.2)
All diabetes peripheral pulses record[c]	Introduction	38.3 (30.6 to 45.9)	7.2 (1.8 to 12.7)	11.0 (-6.8 to 28.8)	-6.6 (-12.3 to -0.9)
All diabetes neuropathy test record	Introduction	0.2 (-1.9 to 2.3)	1.5 (0.8 to 2.2)	58.3 (53.4 to 63.1)	3.2 (0.03 to 6.3)
All diabetes microalbuminuria record	Introduction	1.6 (-1.4 to 4.6)	5.8 (4.8 to 6.9)	28.1 (21.5 to 34.7)	-4.6 (-8.8 to -0.3)
All diabetes HbA1c≤7.4%	Introduction	45.7 (44.7 to 46.6)	2.3 (2.0 to 2.7)	0.2 (-1.8 to 2.1)	-0.5 (-1.7 to 0.7)
All diabetes HbA1c≤10%	Introduction	88.8 (87.5 to 90.0)	1.2 (0.7 to 1.6)	-0.8 (-1.7 to 0.1)	-1.1 (87.5 to 90.0)
All diabetes BP≤145/85mmHg	Introduction	3.5 (3.5 to 3.6)	5.3 (4.2 to 6.5)	5.3 (4.2 to 6.5)	-0.6 (-1.4 to 0.2)
All diabetes cholesterol <5mmol/l	Introduction	44.7 (41.7 to 47.7)	5.7 (4.6 to 6.7)	7.1 (4.6 to 9.6)	-2.3 (-3.7 to -0.9)
All diabetes ACEI/ARB treatment in microalbuminuria	Introduction	71.9 (69.8 to 74.0)	3.7 (3.0 to 4.4)	-6.1 (-7.5 to -4.7)	-2.9 (-3.6 to -2.2)
All diabetes flu immunisation record	Introduction	65.0 (62.8 to 67.3)	1.2 (0.4 to 2.0)	6.9 (1.9 to 11.8)	-0.3 (-3.5 to 2.9)
Matias, 2024 – new ITSA model[b]					
Serious mental illness BMI record	Withdrawal	61.1 (51.8 to 70.4)	1.3 (-4.8 to 7.4)	-16.0 (-30.5 to -1.5)	-1.6 (-7.9 to 4.6)
Serious mental illness cholesterol record (Matias)	Withdrawal	52.5 (42.9 to 62.1)	0.5 (-5.8 to 6.8)	-10.0 (-24.7 to 4.8)	0.1 (-6.3 to 6.5)
Pasvol, 2022 - new ITSA model[b]					
LARC prescribing rate[d]	Introduction	4.8 (4.8 to 4.9)	0.1 (0.1 to 0.2)	0.6 (0.01 to 1.2)	0.1 (-0.1 to 0.3)
LARC prescribing rate[d e]	Withdrawal	-	-	-0.3 (-0.6 to 0.1)	-0.3 (-0.5 to -0.1)
Simpson, 2011[b]					
Hypertension BP record	Introduction	43.0 (31.6 to 54.5)	13.7 (6.2 to 21.2)	5.8 (-11.9 to 23.5)	-12.6 (-20.5 to -4.8)

Hypertension BP≤140/90mmHg	Introduction	37.0 (34.9 to 39.1)	4.0 (2.7 to 5.4)	-1.1 (-4.3 to 2.2)	0.8 (-0.7 to 2.2)
Hypertension BP≤150/90mmHg	Introduction	58.2 (55.0 to 61.4)	4.4 (2.3 to 6.5)	1.1 (-4.5 to 6.7)	-0.7 (-3.5 to 2.1)
Taggar (2012) - new ITSA model[b]					
All patients smoking record	Introduction	17.5 (14.9 to 20.0)	4.3 (3.2 to 5.5)	13.4 (2.6 to 24.1)	0.3 (-3.4 to 4.1)
Chronic condition smoking status record	Introduction	27.9 (26.7 to 29.1)	7.2 (6.6 to 7.9)	22.6 (15.2 to 29.9)	-3.3 (-5.6 to -1.0)
Chronic condition smoking cessation advice record	Introduction	24.9 (18.1 to 31. 7)	8.5 (4.7 to 12.3)	24.1 (7.7 to 40.5)	-5.9 (18.1 to 31.7)

ACEI/ARB: Angiotensin Converting Enzyme Inhibitor/Angiotensin Receptor Blocker; BMI: Body mass index; BP: blood pressure; eGFR: Estimated glomerular filtration rate; ITSA: Interrupted time series analysis; LARC: long-acting reversible contraception

a. Data extracted from table in original paper

b. Data extracted from plot in original paper

c. Model omitting 2003/4 wouldn't converge, fitted with 2003/4 in pre-intervention period

d. LARC prescribing is not specifically incentivised, but giving of LARC advice is with the intention of increasing LARC prescribing

e. Withdrawal fitted as second interruption so follows from introduction model

Supplementary Table E: Impact at one year and three years on quality of care indicators (difference between predicted based on prior trend, and estimated from model given step-change and change in trend)

**Study and indicator**	**Incentive introduction/ withdrawal, or never incentivised**	**Indicator type**	**Impact at one year Percentage point change (95% CI)**	**Impact at three years Percentage point change (95% CI)**
Arrowsmith, 2014 - new ITSA model[b]				
LARC prescribing rate	Introduction	Treatment	0.1 (0.04 to 0.2)	0.4 (0.3 to 0.6)
Calvert, 2009 - new ITSA model[a]				
Type 1 diabetes BMI record	Introduction	Simple process	15.4 (-7.9 to 38.6)	-3.7 (-45.2 to 37.9)
Type 1 diabetes smoking status record	Introduction	Simple process	6.6 (-6.7 to 19.8)	-6.8 (-30.1 to 16.8)
Type 1 diabetes HbA1c record	Introduction	Simple process	3.0 (-8.3 to 14.3)	-13.2 (-33.4 to 7.0)
Type 1 diabetes BP record	Introduction	Simple process	4.0 (2.0 to 6.1)	-7.3 (-10.9 to -3.6)
Type 1 diabetes eGFR record	Introduction	Simple process	8.4 (-4.2 to 20.9)	-6.0 (-28.1 to 16.1)
Type 1 diabetes cholesterol record	Introduction	Simple process	7.4 (-3.6 to 18.4)	-7.1 (-26.6 to 12.4)
Type 1 diabetes smoking cessation advice record	Introduction	Complex process	21.8 (-29.2 to 71.8)	-1.9 (-91.4 to 87.6)
Type 1 diabetes retinopathy screening record	Introduction	Complex process	9.8 (-18.9 to 38.4)	-8.8 (-60.2 to 42.5)
Type 1 diabetes peripheral pulses record	Introduction	Complex process	33.0 (3.3 to 62.8)	17.6 (-34.3 to 69.6)
Type 1 diabetes neuropathy test record	Introduction	Complex process	35.8 (5.9 to 65.8)	21.7 (-30.4 to 73.8)
Type 1 diabetes microalbuminuria record	Introduction	Complex process	24.5 (8.6 to 40.3)	17.0 (-10.8 to 44.8)
Type 1 diabetes cholesterol ≤5mmol/l	Introduction	Intermediate outcome	8.2 (0.2 to 16.2)	0.2 (-13.6 to 14.0)
Type 1 diabetes HbA1c≤7.5%	Introduction	Intermediate outcome	2.3 (-3.8 to 8.3)	-0.7 (-10.7 to 9.2)
Type 1 diabetes HbA1c≤10%	Introduction	Intermediate outcome	3.0 (-7.2 to 13.2)	-11.0 (-29.3 to 7.3)
Type 1 diabetes BP≤145/85mmHg	Introduction	Intermediate outcome	6.1 (3.2 to 9.1)	-0.7 (-4.1 to 2.8)
Type 1 diabetes ACEI/ARB for microalbuminuria	Introduction	Treatment	9.5 (6.1 to 12.8)	19.9 (14.0 to 25.8)
Type 1 diabetes flu immunisation record	Introduction	Treatment	15.6 (0.0 to 31.1)	16.2 (-11.0 to 43.3)
Type 2 diabetes BMI record	Introduction	Simple process	7.2 (-11.7 to 26.1)	Not estimated[d]
Type 2 diabetes smoking status record	Introduction	Simple process	3.8 (-5.7 to 13.2)	Not estimated[d]
Type 2 diabetes HbA1c record	Introduction	Simple process	-0.3 (-5.8 to 5.3)	Not estimated[d]
Type 2 diabetes BP record	Introduction	Simple process	0.9 (-0.3 to 2.2)	-4.6 (-6.8 to -2.3)
Type 2 diabetes eGFR record	Introduction	Simple process	3.5 (-5.5 to 12.6)	Not estimated[d]
Type 2 diabetes cholesterol record	Introduction	Simple process	1.8 (-3.2 to 6.8)	Not estimated[d]
Type 2 diabetes smoking cessation advice record	Introduction	Complex process	14.7 (-21.9 to 51.4)	Not estimated[d]
Type 2 diabetes retinopathy screening record	Introduction	Complex process	6.0 (-7.4 to 19.4)	-8.5 (-31.9 to 14.9)
Type 2 diabetes peripheral pulses record	Introduction	Complex process	26.5 (-3.4 to 56.4)	7.7 (-45.3 to 60.7)
Type 2 diabetes neuropathy test record	Introduction	Complex process	30.3 (-2.2 to 62.8)	10.1 (-47.4 to 67.6)

Type 2 diabetes microalbuminuria record	Introduction	Complex process	22.1 (10.9 to 33.3)	8.5 (-8,5 to 25.4)
Type 2 diabetes HbA1c≤7.5%	Introduction	Intermediate outcome	1.3 (-0.8 to 3.3)	-5.5 (-8.5 to -2.6)
Type 2 diabetes HbA1c≤10%	Introduction	Intermediate outcome	-0.3 (-5.1 to 4.4)	-13.0 (-21.4 to -4.5)
Type 2 diabetes BP≤145/85mmHg	Introduction	Intermediate outcome	5.2 (4.0 to 6.3)	0.7 (-1.2 to 2.6)
Type 2 diabetes cholesterol ≤5mmol/l	Introduction	Intermediate outcome	5.1 (-1.0 to 11.3)	-6.5 (-17.2 to 4.3)
Type 2 diabetes ACEI/ARB for microalbuminuria	Introduction	Treatment	-1.3 (-7.1 to 4.5)	11.1 (-20.7 to -1.6)
Type 2 diabetes flu immunisation record	Introduction	Treatment	11.3 (-4.3 to 26.9)	6.7 (-21.4 to 34.9)
Doran, 2011 - reported by original study				
Asthma smoking status record	Introduction	Simple process	35.6 (33.9 to 37.1)	19.7 (18.0 to 21.3)
Hypertension smoking status record	Introduction	Simple process	30.1 (28.0 to 32.0)	14.0 (11.9 to 15.9)
Hypertension BP record	Introduction	Simple process	3.7 (3.2 to 4.2)	1.7 (1.3 to 2.0)
CHD smoking status record	Introduction	Simple process	16.4 (14.9 to 17.7)	0.7 (−0.9 to 2.1)
CHD BP record	Introduction	Simple process	3.1 (2.6 to 3.6)	0.5 (0.0 to 0.9)
CHD cholesterol record	Introduction	Simple process	5.8 (4.5 to 6.9)	−2.1 (−3.2 to −1.0)
COPD smoking status record	Introduction	Simple process	31.7 (30.9 to 32.4)	16.4 (15.7 to 17.1)
All diabetes HbA1c record	Introduction	Simple process	1.6 (1.0 to 2.2)	−1.5 (−2.2 to −0.9)
All diabetes BP record	Introduction	Simple process	1.6 (1.2 to 1.9)	−0.1 (−0.5 to 0.3)
All diabetes creatinine record	Introduction	Simple process	2.7 (1.9 to 3.4)	−2.1 (−2.7 to −1.5)
All diabetes cholesterol record	Introduction	Simple process	2.0 (1.3 to 2.6)	−2.5 (−3.2 to −1.9)
Lithium prescribed, lithium level record	Introduction	Simple process	13.9 (11.7 to 16.0)	5.3 (3.5 to 7.0)
Lithium prescribed, creatinine and TSH record	Introduction	Simple process	21.1 (18.9 to 23.0)	6.9 (5.4 to 8.3)
Stroke/TIA smoking status record	Introduction	Simple process	37.7 (36.1 to 39.1)	15.2 (13.3 to 16.9)
Stroke/TIA BP record	Introduction	Simple process	6.8 (6.2 to 7.5)	2.6 (2.1 to 3.1)
Stroke/TIA cholesterol record	Introduction	Simple process	14.5 (12.7 to 16.1)	2.0 (0.8 to 3.1)
Hypothyroidism thyroid function record	Introduction	Simple process	3.4 (2.6 to 4.0)	−1.3 (−2.0 to −0.7)
CHD beta-blocker treatment	Introduction	Treatment	2.0 (0.4 to 3.5)	−2.0 (−3.5 to −0.5)
CHD flu immunisation record	Introduction	Treatment	3.9 (2.7 to 5.1)	2.6 (1.4 to 3.8)
CHD and LVSD and ACEI/ARB treatment	Introduction	Treatment	1.2 (−0.9 to 3.1)	−0.4 (−2.4 to 1.3)
COPD flu immunisation record	Introduction	Treatment	8.3 (6.7 to 9.8)	10.4 (8.9 to 11.8)
All diabetes flu immunisation record	Introduction	Treatment	6.3 (5.0 to 7.7)	6.4 (4.9 to 7.8)
Stroke/TIA flu immunisation record	Introduction	Treatment	3.8 (2.2 to 5.4)	3.2 (1.6 to 4.7)
Adults 25-44 years BP record	Never incentivised	Simple process	1.5 (0.3 to 2.8)	0.2 (−1.7 to 2.0)
Peripheral arterial disease cholesterol record	Never incentivised	Simple process	1.6 (−2.8 to 6.1)	−9.5 (−13.7 to −5.3)
Women 50+ and depression, thyroid function record	Never incentivised	Simple process	−0.1 (−0.2 to 0.1)	−0.3 (−0.4 to −0.1)
Dementia thyroid function record	Never incentivised	Simple process	−0.9 (−3.0 to 1.3)	−6.4 (−8.6 to −3.9)

Osteoarthritis weight or BMI record	Never incentivised	Simple process	6.0 (3.6 to 8.4)	11.6 (9.0 to 13.9)
CHD blood sugar record	Never incentivised	Simple process	−1.3 (−3.5 to 0.7)	−5.0 (−6.7 to −3.6)
Hypertension blood sugar record	Never incentivised	Simple process	−3.3 (−5.6 to −1.1)	−9.9 (−11.7 to −8.1)
Hypertension cholesterol record	Never incentivised	Simple process	1.4 (−0.3 to 3.0)	−2.1 (−3.6 to −0.8)
Hypertension creatinine record	Never incentivised	Simple process	0.3 (−1.3 to 1.8)	−4.3 (−5.2 to −3.5)
Congestive heart failure flu immunisation record	Never incentivised	Treatment	−3.1 (−6.7 to 0.3)	−5.2 (-8.9 to −1.8)[i]
Chronic renal disease flu immunisation record	Never incentivised	Treatment	1.7 (−3.5 to 6.6)	−1.9 (−7.1 to 3.1)
Asthma beta-blocker prescription[h]	Never incentivised	Treatment	−0.1 (−0.3 to 0.1)	−0.08 (−0.3 to 0.2)
Diabetes 65+ long acting sulfonylurea prescription[h]	Never incentivised	Treatment	−0.3 (−0.5 to −0.1)	−0.3 (−0.6 to −0.2)
Osteoporosis with relevant treatment	Never incentivised	Treatment	−6.8 (−8.8 to −4.7)	−12.8 (−14.9 to −10.9)
Otitis media with oral decongestant treatment	Never incentivised	Treatment	0.2 (−0.1 to 0.5)	−0.1 (−0.5 to 0.2)
Angina with sumatriptan treatment[h]	Never incentivised	Treatment	−0.01 (−0.5 to 0.5)	0.3 (−0.3 to 0.8)
Back pain with strong analgesic treatment[h]	Never incentivised	Treatment	1.3 (0.0 to 2.6)	0.7 (−0.8 to 2.2)
URTI and similar with antibiotic treatment[h]	Never incentivised	Treatment	4.0 (2.4 to 5.7)	8.2 (6.1 to 10.3)
Splenectomy pneumococcal immunisation record	Never incentivised	Treatment	−3.8 (−6.9 to −0.5)	−5.8 (−9.9 to −1.3)
Kontopantelis, 2013 - new ITSA model[a]				
All diabetes BMI record	Introduction	Simple process	8.5 (7.4 to 9.7)	2.4 (0.6 to 4.3)
All diabetes smoking status record	Introduction	Simple process	8.7 (8.6 to 8.8)	3.3 (1.2 to 5.3)
All diabetes HbA1c record	Introduction	Simple process	-1.7 (-3.1 to -0.4)	Not estimated[d]
All diabetes blood pressure record	Introduction	Simple process	0.1 (-0.4 to 0.7)	Not estimated[d]
All diabetes creatinine record	Introduction	Simple process	-2.7 (-4.4 to 3.8)	Not estimated[d]
All diabetes cholesterol record	Introduction	Simple process	-1.5 (-2.7 to -0.4)	Not estimated[d]
All diabetes smoking advice record	Introduction	Complex process	61.7 (58.2 to 65.3)	59.6 (60.0 to 63.3)
All diabetes retinopathy screening record	Introduction	Complex process	4.0 (0.6 to 7.5)	-14.7 (-19.5 to -9.9)
All diabetes peripheral pulses record[e]	Introduction	Complex process	11.0 (-6.8 to 28.8)	-2.2 (-30.5 to 26.1)
All diabetes neuropathy test record	Introduction	Complex process	58.3 (53.4 to 63.1)	64.6 (59.2 to 69.9)
All diabetes microalbuminuria record	Introduction	Complex process	28.1 (21.5 to 34.7)	19.0 (11.6 to 26.4)
All diabetes HbA1c≤7.4%	Introduction	Intermediate outcome	0.2 (-1.8 to 2.1)	-0.8 (-3.0 to 1.4)
All diabetes HbA1c≤10%	Introduction	Intermediate outcome	-0.8 (-1.7 to 0.1)	-3.0 (-4.7 to 1.3)
All diabetes BP≤145/85mmHg	Introduction	Intermediate outcome	5.3 (4.2 to 6.5)	4.1 (2.9 to 5.3)
All diabetes cholesterol <5mmol/l	Introduction	Intermediate outcome	7.1 (4.6 to 9.6)	2.5 (-1.7 to 6.6)
All diabetes ACEI/ARB treatment in microalbuminuria	Introduction	Treatment	-6.1 (-7.5 to -4.7)	-11.9 (-14.6 to -9.3)
All diabetes flu immunisation record	Introduction	Treatment	6.9 (1.9 to 11.8)	6.3 (0.7 to 11.8)
Ma, 2020 - reported by original study				
LARC prescribing[c f]	Introduction	Treatment	0.54 (0.53 to 0.55)	0.45

Matias, 2024 – new ITSA model[b]				
Serious mental illness BMI record	Withdrawal	Simple process	-16.0 (-30.5 to -1.5)	-19.3 (-44.7 to 6.2)
Serious mental illness cholesterol record (Matias)	Withdrawal	Simple process	-10.9 (-24.7 to 4.8)	-9.8 (-36.0 to 16.4)
Minchin, 2016 – reported by original study				
All adults smoking record	Withdrawal	Simple process	−5.8 (−8.9 to −2.7)	−11.7 (−16.3 to −7.1)
Serious mental illness HbA1c record	Withdrawal	Simple process	−28.5 (−39.0 to −18.0)	−37.5 (−55.4 to −19.6)
Serious mental illness BMI record (Minchin)	Withdrawal	Simple process	−30.2 (−35.5 to −24.9)	−29.9 (−38.9 to −20.9)
Hypothyroidism thyroid function record	Withdrawal	Simple process	−12.1 (−13.6 to −10.6)	−9.2 (−11.4 to −7.0)
Stroke/TIA cholesterol record	Withdrawal	Simple process	−19.8 (−22.4 to −17.2)	−18.0 (−21.8 to −14.2)
All diabetes retinopathy screening record	Withdrawal	Complex process	−11.5 (−16.7 to −6.3)	−14.9 (−22.7 to −7.1)
Epilepsy documentation of being seizure free	Withdrawal	Complex process	−48.7 (−51.8 to −45.6)	−53.6 (−58.2 to −49.0)
Hypertension lifestyle counselling record	Withdrawal	Complex process	−62.3 (−65.6 to −59.0)	−71.6 (−76.5 to −66.7)
Epilepsy preconceptual advice record	Withdrawal	Complex process	−60.5 (−63.5 to −57.5)	−65.9 (−71.0 to −60.8)
LARC advice record	Withdrawal	Complex process	−36.6 (−39.9 to −33.3)	−46.1 (−51.8 to −45.6)
Stroke/TIA cholesterol ≤5mmol/l	Withdrawal	Intermediate outcome	−15.9 (−17.1 to −14.7)	−16.8 (−18.0 to −15.0)
CHD cholesterol ≤5mmol/l	Withdrawal	Intermediate outcome	−10.7 (−13.6 to −7.8)	−10.1 (−14.3 to −5.9)
Morales, 2023 - reported by original study[g]				
Serious mental illness care planning record	Withdrawal	Complex process	−31.0 (−35.0 to −27.1)	−40.2 (−45.5 to −35.0)
All diabetes foot screening record	Withdrawal	Complex process	−13.8 (−20.4 to −7.2)	−22.8 (−33.9 to −11.7)
Peripheral arterial disease BP≤150/90mmHg	Withdrawal	Intermediate outcome	−12.5 (−15.6 to −9.4)	−18.5 (−22.1 to −14.9)
Stroke/TIA BP≤150/90mmHg	Withdrawal	Intermediate outcome	−10.2 (-13.0 to −7.4)[i]	−16.6 (−20.6 to −12.7)
Hypertension BP≤150/90mmHg	Withdrawal	Intermediate outcome	−10.5 (−14.3 to −6.8)	−13.7 (−19.4 to −7.9)
CHD BP≤150/90mmHg	Withdrawal	Intermediate outcome	−8.0 (−9.7 to −6.3)	−12.8 (−14.9 to −10.8)
All diabetes BP≤150/90mmHg	Withdrawal	Intermediate outcome	−6.2 (−8.2 to −4.1)	−10.4 (−13.0 to −7.8)
All diabetes BP≤140/80mmHg	Withdrawal	Intermediate outcome	−7.8 (−10.1 to −5.6)	−12.7 (−15.0 to −10.4)
All diabetes HbA1c≤75mmol/mol	Withdrawal	Intermediate outcome	−3.2 (−5.4 to −0.9)	−5.0 (−8.4 to −1.5)
All diabetes HbA1c≤64mmol/mol	Withdrawal	Intermediate outcome	−2.4 (−4.8 to −0.05)	−3.4 (−6.7 to −0.03)
All diabetes HbA1c≤59mmol/mol	Withdrawal	Intermediate outcome	−1.9 (−4.5 to 0.8)	−2.1 (−5.7 to 1.6)
Stroke/TIA flu immunisation record	Withdrawal	Treatment	−3.9 (−6.9 to −0.9)	−3.9 (−7.8 to 0.1)
COPD flu immunisation record	Withdrawal	Treatment	−3.8 (−6.9 to −0.8)	−3.4 (−7.3 to 0.4)
CHD flu immunisation record	Withdrawal	Treatment	−3.2 (−6.3 to 0.03)	−3.2 (−7.6 to 1.2)
All diabetes flu immunisation record	Withdrawal	Treatment	−3.3 (−6.9 to 0.2)	−2.4 (−7.2 to 2.5)
CHD antiplatelet or anticoagulant treatment	Withdrawal	Treatment	−0.8 (−1.8 to 0.3)	−1.4 (−3.3 to 0.6)
Pasvol, 2022 - new ITSA model[b]				
LARC prescribing rate[c]	Introduction	Treatment	0.6 (0.01 to 1.2)	0.8 (0.5 to 1.1)

LARC prescribing rate[c]	Withdrawal	Treatment	-0.3 (-0.6 to 0.1)	-0.8 (-1.5 to -0.1)
Simpson, 2011 – new ITSA model[b]				
Hypertension BP record	Introduction	Simple process	5.8 (-11.9 to 23.5)	Not estimated[d]
Hypertension BP≤140/90mmHg	Introduction	Intermediate outcome	-1.1 (-4.3 to 2.2)	0.5 (-5.2 to 6.2)
Hypertension BP≤150/90mmHg	Introduction	Intermediate outcome	1.1 (-4.5 to 6.7)	-0.3 (-9.5 to 8.9)
Taggar, 2012 - new ITSA model[b]				
All patients smoking status record	Introduction	Simple process	13.4 (2.6 to 24.1)	14.0 (7.3 to 20.8)
Chronic condition smoking status record	Introduction	Simple process	22.6 (15.2 to 29.9)	15.9 (10.9 to 20.9)
Chronic condition smoking cessation advice record	Introduction	Complex process	24.1 (7.7 to 40.5)	12.3 (-7.5 to 32.1)

ACEI/ARB: Angiotensin Converting Enzyme Inhibitor/Angiotensin Receptor Blocker; BMI: Body mass index; BP: blood pressure; CHD: Coronary heart disease; COPD: Chronic obstructive pulmonary disease; eGFR: Estimated glomerular filtration rate; ITSA: Interrupted time series analysis; LARC: long-acting reversible contraception; LVSD: left ventricular systolic dysfunction; TIA: transient ischaemic attack; TSH: thyroid stimulating hormone

a. Data extracted from table in original paper
b. Data extracted from plot in original paper
c. LARC prescribing is not specifically incentivised, but giving of LARC advice is with the intention of increasing LARC prescribing
d. Not estimated because predicted value based on prior trend exceeds 100%
e. Model omitting 2003/4 wouldn't converge, fitted with 2003/4 in pre-intervention period
f. Paper reports change after four years without confidence interval ; assumed to be not significant
g. Estimated change of withdrawal in Scotland controlled for changes in England where incentives were maintained
h. Reversed indicators (lower is better, but analysed in reverse so that higher is better)
i. Original paper reported 95% CI with no negative sign before lower bound, assumed to be an error and corrected

Supplementary Table F: Matched indicators with one and three year impact data for both incentive introduction and incentive withdrawal

Indicator group name in Figure 3	Indicator for incentive introduction	Indicator for incentive withdrawal	One year impact of introduction* (percentage point, 95% CI)	One year impact of withdrawal* (percentage point, 95% CI)	3 year impact of introduction* (percentage point, 95% CI)	3 year impact of withdrawal* (percentage point, 95% CI)
Stroke/TIA cholesterol record	Cholesterol record in stroke/TIA[7]	Cholesterol record in stroke/TIA[22]	14.5 (12.7 to 16.1)	-19.8 (-22.4 to -17.2)	2.0 (0.8 to 3.1)	-18.0 (-21.8 to -14.2)
All adults smoking status record	All patients smoking status record[30]	All adults smoking record[22]	13.4 (2.6 to 24.1)	-5.8 (-8.9 to -2.7)	14.0 (7.3 to 20.8)	-11.7 (-16.3 to -7.1)
COPD flu immunisation	COPD flu immunisation[7]	COPD flu immunisation[23]	8.3 (6.7 to 9.8)	-3.8 (-6.9 to -0.8)	10.4 (8.9 to 11.8)	-3.4 (-7.3 to 0.4)
Diabetes flu immunisation	All diabetes flu immunisation[7]<br>All diabetes flu immunisation[17]	All diabetes flu immunisation[23]	6.3 (5.0 to 7.7)<br>6.9 (1.9 to 11.8)	-3.3 (-6.9 to 0.2)	6.4 (4.9 to 7.8)<br>6.3 (0.7 to 11.8)	-2.4 (-7.2 to 2.5)
Diabetes BP control	All diabetes BP≤145/85mmHg[17]	All diabetes BP≤140/80mmHg[23]<br>All diabetes BP≤150/90mmHg[23]	5.3 (4.2 to 6.5)	-7.8 (-10.1 to -5.6)<br>-6.2 (-8.2 to -4.1)	4.1 (2.9 to 5.3)	-12.7 (-15.0 to -10.4)<br>-10.4 (-13.0 to -7.8)
Diabetes retinal screen	All diabetes retinal screening record[17]	All diabetes, retinal screening record[22]	4.0 (0.6 to 7.5)	-11.5 (-16.7 to -6.3)	-14.7 (-19.5 to -9.9)	-14.9 (-22.7 to -7.1)
Stroke/TIA flu immunisation	Stroke/TIA flu immunisation[7]	Stroke/TIA flu immunisation[23]	3.8 (2.2 to 5.4)	-3.9 (-6.9 to -0.9)	3.2 (1.6 to 4.7)	-3.9 (-7.8 to 0.1)
CHD flu immunisation	CHD flu immunisation[7]	CHD flu immunisation[23]	3.9 (2.7 to 5.1)	-3.2 (-0.2 to 1.4)	2.6 (1.4 to 3.8)	-3.2 (-7.6 to 1.2)
Hypothyroid monitoring	Hypothyroidism thyroid function record[7]	Hypothyroidism thyroid function record[22]	3.4 (2.6 to 4.0)	−12.1 (−13.6 to −10.6)	-1.3 (-2.0 to -0.7)	−9.2 (−11.4 to −7.0)
CHD secondary prevention drugs	CHD and LVSD ACEI/ARB treatment[7]<br>CHD beta-blocker treatment[7]	CHD antithrombotic treatment[23]	1.2 (-0.9 to 3.1)<br>2.0 (0.4 to 3.5)	-0.8 (-1.8 to 0.3)	-0.4 (-2.4 to 1.3)<br>-2.0 (-3.5 to -0.5)	-1.4 (-3.3 to 0.6)
Hypertension BP≤150/90mmHg	Hypertension BP≤150/90[27]	Hypertension BP≤150/90[22]	1.1 (-4.5 to 6.7)	-10.5 (-14.3 to -6.8)	0.3 (-9.5 to 8.9)	-13.7 (-19.4 to -7.9)
LARC prescribing	LARC prescribing rate[24]	LARC prescribing rate[24]	0.6 (0.01 to 1.2)	-0.3 (-0.6 to 0.1)	0.8 (0.5 to 1.1)	-0.8 (-1.5 to -0.1)
Diabetes HbA1c≤7.5%	All diabetes HbA1c≤7.4%[17]	All diabetes HbA1c≤59mmol/mol[23 #]	0.2 (-1.8 to 2.1)	-1.9 (-4.5 to 0.8)	-0.8 (-3.0 to 1.4)	-2.1 (-5.7 to 1.6)
Diabetes HbA1c≤10% or 9%	All diabetes HbA1c≤10%[17]	All diabetes HbA1c≤75mmol/mol[23 #]	-0.8 (-1.7 to 0.1)	-3.2 (-5.4 to -0.9)	-3.0 (-4.7 to 1.3)	-5.0 (-8.4 to -1.5)

ACEI: Angiotensin Converting Enzyme Inhibitor; ARB: Angiotensin Receptor Blocker; BP: Blood pressure; CHD: Coronary heart disease; CI: Confidence interval; COPD: Chronic obstructive pulmonary disease; LARC: Long-acting reversible contraception prescribing; LVSD: Left ventricular systolic dysfunction; T1 diabetes: Type 1 diabetes; T2 diabetes: Type 2 diabetes; TFT: thyroid function testing; TIA: transient ischaemic attack

* Positive change = higher observed quality than predicted by prior trend; negative change = lower observed quality than predicted by prior trend

75mmol/mol = 9%; 59mmol/mol = 7.5%

Supplementary Table G: Summary of findings of studies included in the narrative synthesis

Study	Intervention examined	Summary of findings
Campbell (2009)	Incentive introduction	Two timepoints before and two after based on manual review of individual patient's health records. Composite health record review measures of both incentivised and non-incentivised quality of care in asthma, diabetes, and coronary heart disease; composite patient survey measures of access, interpersonal care and continuity (none directly incentivised by QOF, although QOF in April 2004 did incentivise use of patient experience measures by practices and some aspects of speed of access). Immediately after QOF introduction in April 2004 (measurement in 2005), quality improved faster than expected for asthma and diabetes, but not coronary heart disease. Quality then plateaued at a high level for all conditions (measurement in 2007). Patient evaluation of interpersonal care did not change, rapid access improved, but access to a particular doctor and continuity got worse. For asthma and coronary heart disease, incentivised care improved more than non-incentivised, but changes were similar in both for diabetes.
Fichera (2016)	Incentive introduction	Health Survey for England repeated cross-sectional data 1997-2009. Sharp regression discontinuity design at time of QOF implementation in April 2004 (including incentives to record lifestyle risk factors in people with various chronic conditions) for mean number of cigarettes smoked per day, mean body mass index, and proportion drinking alcohol every day. Statistically significant reduction in mean number of cigarettes smoked per day (of 0.7 cigarettes per day; ~18% reduction in mean cigarettes), but not in the other outcomes.
Gallagher (2015)	Incentive introduction	Individual patient data analysis 1999-2008 of initiation of pharmacological treatment of type 2 diabetes in the 12 months after diagnosis. Before QOF incentivisation in April 2004 of multiple diabetes indicators including indicators for HbA1c measurement and control, the proportion initiated within 12 months of diagnosis was falling by 1.2 (95% CI -2.0 to -0.5) percentage points per year, changing to an increase after incentivisation by 1.9 (95%CI 1.1 to 2.7) percentage points per year.
Gilbert (2019)	Incentive introduction	Individual patient data analysis 2000/1 to 2014/15 examining total consultation rates with GPs and practice nurses. Mean total GP consultation rates were declining 2000/1 to 2003/4 (by 0.445 consultations per year), with a significant change in trend with QOF introduction (0.463) to a flat post-QOF introduction trend. Mean total nurse consultations were increasing 2000/1 to 2003/4 (by 0.321 consultations per year), with a significant step change with QOF introduction (0.321), and a change in trend (-0.363) to a flat trend post-QOF introduction. For all types of consultation, consultation rates were flat before QOF introduction (-0.121, p=0.331), with no step change (0.328, p=0.265) or change in trend (0.096, p=0.442)
Hardoon (2016)	Incentive introduction	Individual patient data analysis 2000 to 2013 of alcohol consumption recording in people with bipolar disorder compared to people without serious mental illness, analysis in two year periods (two before any QOF incentivisation – 2000-2002, 2002-2004), one after QOF incentivisation of 'annual review' (2004-2006), two after QOF incentivisation of 'lifestyle screening' (2007-2009, 2009-2011), and one after QOF incentivisation of alcohol screening (2011-2013). The adjusted rate ratios for alcohol recording in people with bipolar vs people without serious mental illness were 0.96 (95% CI 0.88-0.95) and 1.09 (1.04-1.16) before any incentivisation, 1.39 (1.31-1.47) after 'annual review' incentivisation, 1.89 (1.77-2.01) and 2.22 (2.09-2.36) after 'lifestyle review' incentivisation, and 4.45 (4.15-4.77) after alcohol screening incentivisation.
Harrison (2014)	Incentive introduction	National individual patient data analysis 1998/9 to 2010/11 examining changes associated with QOF implementation in April 2004 in 'incentivised' ambulatory care sensitive conditions (ACSC) admissions compared to 'non-incentivised ACSC admissions and non-ACSC admissions. Compared to non-incentivised ACSC admissions, trend-adjusted incentivised ACSC admission rates were -2.68% (95%CI -3.79 to -1.57) lower in the year after QOF implementation (2004/5) and -7.66 (-8.78 to -6.55) percentage points lower three years after (2006/7). Compared to non-ACSC admissions, trend-adjusted incentivised ACSC admission rates were -2.78% (95%CI -3.58 to -1.99) lower

		in the year after QOF implementation (2004/5) and -8.65 (--9.45 to –10.53) percentage points lower three years after (2006/7).
James (2014)	Incentive introduction	Individual patient data analysis 2000 to 2009, finding that COPD prevalence increased steadily over the period, with steady reductions in COPD severity (consistent with earlier diagnosis), and no apparent discontinuity with QOF COPD incentives' introduction in April 2004. Consultation rates in people with COPD were stable 2000-2002, increased in 2003 (the QOF preparatory year) and again in 2004 (first year of QOF implementation) – 9.4 consultations per patient per year in 2000, 10.5 in 2004 – with further increases 2007-2009 (11.3 in 2009).
Kendrick (2015)	Incentive introduction	Individual patient data analysis 2003 to 2013, with the outcome of pharmacological treatment of new and recurrent episodes of depression. Interrupted time-series analysis was used to examine changes associated with new national guidelines in December 2004 and QOF incentivisation in April 2005 of assessment of depression severity at diagnosis using an approved severity measure. For new episodes of depression, both national guidelines and QOF implementation were associated with immediate declines in pharmacological treatment (-4.15 [95%CI -7.26 to -1.03] percentage points in the next quarter for national guidelines, -4.38 [-6.48 to -2.28] for QOF), but no statistically significant changes in trend. There were no significant changes associated with either intervention for pharmacological treatment of recurrent episodes of depression.
Khadjesari (2017)	Incentive introduction	Individual patient data analysis 2000 to 2013 of alcohol consumption recording in people with schizophrenia compared to people without serious mental illness, analysis in two year periods (two before any QOF incentivisation – 2000-2002, 2002-2004), one after QOF incentivisation of 'annual review' (2004-2006), two after QOF incentivisation of 'lifestyle screening' (2007-2009, 2009-2011), and one after QOF incentivisation of alcohol screening (2011-2013). The adjusted rate ratios for alcohol recording in people with schizophrenia vs people without serious mental illness were 0.89 (95% CI 0.84-0.96) and 0.99 (0.95-1.03) before any incentivisation, 1.34 (1.28-1.41) after 'annual review' incentivisation, 1.79 (1.69-1.89) and 2.09 (1.96-2.22) after 'lifestyle review' incentivisation, and 3.80 (3.54-4.08) after alcohol screening incentivisation.
Kontopantelis (2014)	Incentive withdrawal	Individual patient data analysis 2004 to 2012, examining changes associated with withdrawal of incentivisation for two indicators in 2006 (two years of incentivisation – flu immunisation in asthma, and record of recent lithium level for patients prescribed lithium) and six indicators in 2011 (all simple process measures – three for record of blood pressure [in coronary heart disease [CHD], diabetes and stroke], two for record of cholesterol [in CHD and diabetes] and one for record of HbA1c [in diabetes]) with a single year of post-withdrawal follow-up. Lithium measurement remained incentivised by a linked incentivised indicator for lithium in the therapeutic range, and the six simple process indicators remain incentivised by linked intermediate outcome indicators (since 'not recorded' counts as 'fail intermediate outcome target' and clinicians also still received alerts to measure blood pressure/cholesterol/HbA1c as a result).<br><br>Estimated differences six years (2011/12) after incentive removal for flu immunisation in asthma was -0.70 (95%CI -1.01 to -0.39) percentage points, and after incentive removal for lithium record in people prescribed lithium was 0.65 (-0.11 to 1.46) percentage points. Estimated differences one year after (2011/12) removal of incentives for the six simple processes were -.0.02 (95% CI -0.24 to 0.20) percentage points for BP record in CHD, 0.04 (-0.10 to 0.18) percentage points for BP record in diabetes, -0.18 (-0.43 to 0.07) percentage points for BP record in stroke, -1.19 (-1.56 to -0.81) percentage points for cholesterol record in CHD, -0.18 (-0.36 to 0.001) percentage points for cholesterol record in diabetes, and -0.15 (-0.32 to 0.02) for HbA1c record in diabetes.
Kontopantelis (2015)	Incentive introduction	Individual patient data analysis 2000 to 2012 for people with serious mental illness (SMI) linked to five age, sex and general practice matched controls without SMI. Analysis examined changes in consultation rates associated with QOF incentivisation of SMI annual review from April 2004 in people with SMI versus people without SMI. In people with SMI, there was a significant rising trend in face-to-face consultation rates after QOF implementation (0.56 [95%CI 0.02 to 0.36] consultations per patient per year) compared to no change in

		people without SMI. Telephone consultation was much less common, but post-QOF rates increased faster in people with SMI than people without although absolute differences are small.
Matias (2024)	Incentive (re)introduction *and* incentive withdrawal	Individual patient data analysis 2011 to 2020, examining change in recording of body mass index (BMI), cholesterol and alcohol in people with serious mental illness (SMI) response to changing QOF incentives, using difference-in-difference (DiD) analysis controlled for recording of blood pressure in people with SMI which was incentivised throughout. For withdrawal of incentives for BMI record in 2014, the DiD estimate for impact in the first year was -12.3 (95%CI -14.2 to -10.4) percentage points, and in the second year -14.3 (-16.5 to -12.0) percentage points. For withdrawal of incentives for cholesterol record in 2014, the DiD estimate for impact in the first year was -6.8 (95%CI -8.3 to -5.3) percentage points, and the same in the second year. For the reintroduction of incentives for BMI record in 2019, the DiD estimate was 10.2 (6.3 to 14.1) percentage points in the first year. For the withdrawal of incentives for alcohol record in 2019, the DiD estimate was -11.9 (-16.2 to -7.6) percentage points.<br><br>Incentive withdrawal raw data for BMI and cholesterol in 2014 was reanalysed for quantitative synthesis (the two other comparisons did not have enough post-intervention data to reanalyse – single time point).
McLintock (2014)	Incentive introduction	Individual patient data analysis 2002 to 2012, examining new depression diagnoses and new antidepressant prescription in people in whom depression was incentivised in 2006 (diabetes and coronary heart disease) and people with chronic conditions in whom there was no incentivisation (hypertension, epilepsy, chronic obstructive pulmonary disease, asthma). In incentivised patients, depression diagnosis increased from 21/100000 pts/month in 2002-2004 to 94 in 2007-2012 (OR 2.09, 95%CI 1.92 to 2.27) compared to an increase from 27/100000 pts/month to 77 in non-incentivised patients (OR 1.53, 1.46 to 1.62). After incentives were introduced, new antidepressant prescribing was higher in patients with incentivised conditions compared to those with non-incentivised conditions.
Millett (2009)	Incentive introduction	In 2004 (one year after QOF implementation), then the difference between actual and predicted achievement was 1.5 (95%CI 1 to 1.9) percentage points for BP≤140/80mmHg, -1.6 (2.1 to -1.1) percentage points for HbA1c≤7%, and 3 (2.4 to 3.5) percentage points for total cholesterol≤5mmol/L. In 2005 (two years after QOF implementation), then the difference between actual and predicted achievement was 2.5 (95%CI 2.1 to 3) percentage points for BP≤140/80mmHg, -2.4 (-2.9 to -1.9) percentage points for HbA1c≤7%, and 4.9 (4.4 to 5.4) percentage points for total cholesterol≤5mmol/L. People with diabetes with more comorbidities appeared to have more improvement in BP and cholesterol control than those with fewer or none, and less worsening in HbA1c control.
Ryan (2016)	Incentive introduction	Population-level mortality data for UK and other high-income countries 1994-2010, using difference-in-differences (DiD) to examine changes in age-sex adjusted mortality for conditions targeted by QOF (and various secondary outcomes), compared to a 'synthetic UK' weighted combination of other countries. The DiD estimate for the primary outcome was -3.68 (95%CI -8.16 to 0.80) deaths per 100000 population (greater reduction in mortality in the UK than in the synthetic UK comparator, but not statistically significant). Secondary outcomes DiD outcomes were similarly not statistically significantly different (ischaemic heart disease mortality -2.21/100000, cancer 0.28/100000, non-targeted conditions 11.60/100000).
Serumaga (2011)	Incentive introduction	Individual patient data analysis 2002 to 2007, using interrupted time-series analysis to examine measurement and control (BP≤150/90mmHg) of blood pressure and treatment intensity in people with hypertension. The proportion with a BP record increased and the proportion with controlled BP decreased throughout the period studied, despite steady increases in treatment intensity. For BP recorded, the level change at QOF incentive introduction in 2004 was 0.85 (95%CI -3.04 to 4.74) percentage point, and the trend change -0.01 (-0.24 to 0.21) percentage points per quarter. For BP control, the level change was -1.19 (95%CI -2.06 to 1.09) percentage point, and the trend change -0.01 (-0.06 to 0.03) percentage points per quarter. There was no evidence of change in rates of cardiovascular events or

		total mortality.
Sheppard (2018)	Incentive introduction *and* incentive withdrawal	Individual patient data analysis 1998 to 2016, examining monthly incidence of lifestyle advice and antihypertensive drug treatment in people with mild (blood pressure 140/90 to 159/99mmHg) hypertension. Examined interventions were introduction of QOF incentives for lifestyle advice and cardiovascular risk assessment at diagnosis AND introduction of NHS England Health Checks in April 2009, and withdrawal of QOF incentives in April 2013. Primary/pre-specified analysis ignored QOF hypertension incentives from April 2004 onwards for checking and controlling blood pressure, and to record smoking status and give smoking cessation advice to smokers. Interrupted time series analysis found that lifestyle advice incidence rose more quickly from 2009 onwards (potentially confounded by NHS Health Check introduction, and the pre-intervention time-series contains an unmodelled but very visually apparent impact of QOF introduction in 2004), then declined when QOF incentives were changed in 2013. Incidence of antihypertensive drug treatment declined throughout the whole study with no evidence of impact of either intervention. Post-hoc analysis also modelling QOF introduction in 2004 found a step change in lifestyle advice implementation in 2004 but then some decline in incidence to 2009, then a change to a rising incidence after 2009, and a decline after 2013 (but the 2004 intervention ignores visually apparent large change in lifestyle advice in the QOF preparatory year April 2003-Mar 2004). Overall, the analysis provides evidence that QOF incentivisation in 2004 was associated with increased incidence of lifestyle advice (plausibly driven by incentivised advice to stop smoking) but with a waning effect, and that introduction of incentives for broader lifestyle advice in 2009 was associated with rising incidence of risk factor recording, with the impact reversed when broader lifestyle incentives were withdrawn in 2013.
Sutton (2010)	Incentive introduction	Individual patient data analysis 2000 to 2006 examining changes associated with QOF introduction in annual rates of recording blood pressure, smoking status, cholesterol, body mass index and alcohol consumption in both people with conditions targeted by incentives, and people not targeted. Incentivisation was associated with an average 7.9 percentage point increase in risk factor recording in the whole population – 15.4 percentage points in the targeted group and 1.4 percentage points in the untargeted group (so no evidence of crowding out in the untargeted and some evidence of 'halo effects').
Szatkowski (2016)	Incentive introduction	Individual patient data analysis 2004 to 2013 examining change to QOF incentives for smoking record and smoking cessation advice in 2012 (in people with chronic conditions, incentivisation of record of 'support and treatment' rather than 'cessation advice'; in the general population, then new incentives for 'support and treatment' [no prior incentive for 'cessation advice']) using ARIMA modelling of monthly data. In the whole population in 2012-2013, compared to 2010-2012, there were 18.9% (95%CI 9.9 to 27.9) increases in record of cessation advice, 38.1% (19.3 to 57.0) increase in referral to NHS Stop Smoking Services, but a -13.8% (-21.0 to -6.5) decrease in prescription for pharmacotherapy (eg nicotine replacement therapy, which can also be bought over the counter and prescribed outside the practice including by Stop Smoking Services).
Wilson (2017)	Incentive introduction	Individual patient data analysis 1996 to 2014, using interrupted time-series analysis to examine impact of QOF incentivisation of 'annual review' of people with serious mental illness (SMI) 2004-2011, and of recording of specific cardiovascular risk factors in 2011 (ie two interruptions/interventions). Outcomes were annual rates of first ever record of cholesterol≥5mmol/L, first diagnosis of diabetes, first diagnosis/record of obesity, and first diagnosis of hypertension (all 'case-finding'); and first prescription of anti-diabetic medication, and first prescription of lipid-modifying medication (both 'treatment'). Analysis compared rates in people with and without SMI. The study found increases in first record of risk factors or diagnoses in people with SMI (and greater increases than in people without SMI) associated with QOF introduction in 2004, but less change after specific incentivisation in 2011. However, there was no strong evidence of increases in prescribing, and no differences between people with and without SMI.

References

1. Guthrie B, Tang J. What did we learn from 12 years of QOF? http://www.sspc.ac.uk/media/media_486342_en.pdf. Glasgow: Scottish School of Primary Care, 2016.
2. Roland M, Guthrie B. Quality and Outcomes Framework: what have we learnt? *BMJ* 2016; **354**: i4060.
3. Roland M, Olesen F. Can pay for performance improve the quality of primary care? *BMJ* 2016; **354**.
4. Arrowsmith ME, Majeed A, Lee JT, Saxena S. Impact of pay for performance on prescribing of long-acting reversible contraception in primary care: an interrupted time series study. *PloS one* 2014; **9**(4): e92205.
5. Calvert M, Shankar A, McManus RJ, Lester H, Freemantle N. Effect of the quality and outcomes framework on diabetes care in the United Kingdom: retrospective cohort study. *BMJ (Clinical research ed)* 2009; **338**(8900488, bmj, 101090866): b1870.
6. Campbell SM, Reeves D, Kontopantelis E, Sibbald B, Roland M. Effects of Pay for Performance on the Quality of Primary Care in England. *NEJM* 2009; **361**(4): 368-78.
7. Doran T, Kontopantelis E, Valderas JM, et al. Effect of financial incentives on incentivised and non-incentivised clinical activities: Longitudinal analysis of data from the UK Quality and Outcomes Framework. *BMJ* 2011; **343**(7814): d3590.
8. Fichera E, Gray E, Sutton M. How do individuals' health behaviours respond to an increase in the supply of health care? Evidence from a natural experiment. *Social science & medicine (1982)* 2016; **159**(ut9, 8303205): 170-9.
9. Gallagher N, Cardwell C, Hughes C, O'Reilly D. Increase in the pharmacological management of Type 2 diabetes with pay-for-performance in primary care in the UK. *Diabetic medicine : a journal of the British Diabetic Association* 2015; **32**(1): 62-8.
10. Gilbert C, Allgar V, Doran T. Workload impact of the Quality and Outcomes Framework for patients with diabetes: an interrupted time series in general practice. *The British journal of general practice : the journal of the Royal College of General Practitioners* 2019; **69**(685): e570-e7.
11. Hardoon SL, Khadjesari Z, Nazareth I, Hamilton FL, Petersen I. Monitoring of alcohol consumption in primary care among adults with bipolar disorder: A cross-sectional and retrospective cohort study. *Journal of affective disorders* 2016; **198**(h3v, 7906073): 83-7.
12. Harrison MJ, Dusheiko M, Sutton M, Gravelle H, Doran T, Roland M. Effect of a national primary care pay for performance scheme on emergency hospital admissions for ambulatory care sensitive conditions: controlled longitudinal study. *BMJ (Clinical research ed)* 2014; **349**(8900488, bmj, 101090866): g6423.
13. James GD, Donaldson GC, Wedzicha JA, Nazareth I. Trends in management and outcomes of COPD patients in primary care, 2000-2009: a retrospective cohort study. *NPJ primary care respiratory medicine* 2014; **24**(101631999): 14015.
14. Kendrick T, Stuart B, Newell C, Geraghty AWA, Moore M. Did NICE guidelines and the Quality Outcomes Framework change GP antidepressant prescribing in England? Observational study with time trend analyses 2003-2013. *Journal of affective disorders* 2015; **186**(h3v, 7906073): 171-7.
15. Khadjesari Z, Hardoon SL, Petersen I, Hamilton FL, Nazareth I. Impact of Financial Incentives on Alcohol Consumption Recording in Primary Health Care Among Adults with Schizophrenia and Other Psychoses: A Cross-Sectional and Retrospective Cohort Study. *Alcohol and Alcoholism* 2017; **52**(2): 197-205.
16. Kontopantelis E, Olier I, Planner C, et al. Primary care consultation rates among people with and without severe mental illness: a UK cohort study using the Clinical Practice Research Datalink. *BMJ open* 2015; **5**(12): e008650.
17. Kontopantelis E, Reeves D, Valderas JM, Campbell S, Doran T. Recorded quality of primary care for patients with diabetes in England before and after the introduction of a financial incentive scheme: a longitudinal observational study. *BMJ quality & safety* 2013; **22**(1): 53-64.

18. Kontopantelis E, Springate D, Reeves D, Ashcroft DM, Valderas JM, Doran T. Withdrawing performance indicators: retrospective analysis of general practice performance under UK Quality and Outcomes Framework. *BMJ : British Medical Journal* 2014; **348**: g330.
19. Ma R, Cecil E, Bottle A, French R, Saxena S. Impact of a pay-for-performance scheme for long-acting reversible contraceptive (LARC) advice on contraceptive uptake and abortion in British primary care: An interrupted time series study. *PLoS medicine* 2020; **17**(9): e1003333.
20. McLintock K, Russell AM, Alderson SL, et al. The effects of financial incentives for case finding for depression in patients with diabetes and coronary heart disease: interrupted time series analysis. *BMJ open* 2014; **4**(8): e005178.
21. Millett C, Bottle A, Ng A, et al. Pay for perfomance and the quality of diabetes management in individuals with and without co-morbid medical conditions. *Journal of the Royal Society of Medicine* 2009; **102**(9): 369-77.
22. Minchin M, Roland M, Richardson J, Rowark S, Guthrie B. Quality of Care in the United Kingdom after Removal of Financial Incentives. *New England Journal of Medicine* 2018; **379**(10): 948-57.
23. Morales DR, Minchin M, Kontopantelis E, Roland M, Sutton M, Guthrie B. Estimated impact from the withdrawal of primary care financial incentives on selected indicators of quality of care in Scotland: controlled interrupted time series analysis. *Bmj* 2023; **380**: e072098.
24. Pasvol TJ, Macgregor EA, Rait G, Horsfall L. Time trends in contraceptive prescribing in UK primary care 2000-2018: a repeated cross-sectional study. *BMJ sexual & reproductive health* 2022; **48**(3): 193-8.
25. Ryan AM, Krinsky S, Kontopantelis E, Doran T. Long-term evidence for the effect of pay-for-performance in primary care on mortality in the UK: a population study. *Lancet (London, England)* 2016; **388**(10041): 268-74.
26. Sheppard JP, Stevens S, Stevens RJ, et al. Association of guideline and policy changes with incidence of lifestyle advice and treatment for uncomplicated mild hypertension in primary care: a longitudinal cohort study in the Clinical Practice Research Datalink. *BMJ open* 2018; **8**(9): e021827.
27. Simpson CR, Hannaford PC, Ritchie LD, Sheikh A, Williams D. Impact of the pay-for-performance contract and the management of hypertension in Scottish primary care: a 6-year population-based repeated cross-sectional study. *The British journal of general practice : the journal of the Royal College of General Practitioners* 2011; **61**(588): e443-51.
28. Sutton M, Elder R, Guthrie B, Watt G. Record rewards: the effects of targeted quality incentives on the recording of risk factors by primary care providers. *Health economics* 2010; **19**(1): 1-13.
29. Szatkowski L, Aveyard P. Provision of smoking cessation support in UK primary care: impact of the 2012 QOF revision. *The British journal of general practice : the journal of the Royal College of General Practitioners* 2016; **66**(642): e10-5.
30. Taggar JS, Coleman T, Lewis S, Szatkowski L. The impact of the Quality and Outcomes Framework (QOF) on the recording of smoking targets in primary care medical records: cross-sectional analyses from The Health Improvement Network (THIN) database. *BMC public health* 2012; **12**(100968562): 329.
31. Wilson CL, Rhodes KM, Payne RA. Financial incentives improve recognition but not treatment of cardiovascular risk factors in severe mental illness. *PloS one* 2017; **12**(6): e0179392.
